# An Increasing Misalignment Between Crop Distribution and Environmental Resources Under Climate Change in China

**DOI:** 10.1002/advs.202516290

**Published:** 2026-03-02

**Authors:** Zheng'e Su, Zhentao Zhang, Jin Zhao, Xia Liang, Chuang Zhao, Zhijuan Liu, Minghao Zhuang, Jing Sun, Bo Lu, Ke Liu, Johannes W. M. Pullens, Matthew T. Harrison, Deli Chen, Xiaoguang Yang

**Affiliations:** ^1^ College of Resources and Environmental Sciences China Agricultural University Beijing China; ^2^ Sanya Institute of China Agricultural University Sanya China; ^3^ School of Agriculture, Food and Ecosystem Sciences (SAFES), Faculty of Science The University of Melbourne Parkville Victoria Australia; ^4^ State Key Laboratory of Urban and Regional Ecology, Research Center for Eco‐Environmental Sciences Chinese Academy of Sciences Beijing China; ^5^ State Key Laboratory of Efficient Utilization of Arid and Semi‐arid Arable Land in Northern China, Institute of Agricultural Resources and Regional Planning Chinese Academy of Agricultural Sciences Beijing China; ^6^ State Key Laboratory of Climate System Prediction and Risk Management/China Meteorological Administration Climate Studies Key Laboratory, National Climate Centre China Meteorological Administration Beijing China; ^7^ Hebei Key Laboratory of Meteorological Artificial Intelligence/FDU‐XMetAI Joint Lab on Earth System Intellegent Prediction Xiong'an Institute of Meteorological Artificial Intelligence Xiong'an New Area China; ^8^ Collaborative Innovation Center on Forecast and Evaluation of Meteorological Disasters(CIC‐FEMD) Nanjing University of Information Science & Technology Nanjing China; ^9^ Tasmanian Institute of Agriculture University of Tasmania Launceston Tasmania Australia; ^10^ Department of Agroecology Aarhus University Tjele Denmark; ^11^ iCLIMATE ‐ Aarhus University Interdisciplinary Centre for Climate Change Aarhus University Tjele Denmark

**Keywords:** climate change, crop distribution, environmental resources, water availability

## Abstract

Understanding local environmental resources is key to easing resource pressure and achieving sustainable crop production under climate change. Using multi‐source data and a crop model, the integrated environmental resource endowment, encompassing climatic conditions, blue water availability and soil properties, for maize and wheat, and how harvest areas align with these resources is quantified. Over the past 20 years, maize shifted northward with climate changes, while wheat's high endowment regions moved west but its harvest area moved east. Notably, both crops show increasing spatial misalignment with water resources, with about 84% of maize and 90% of wheat areas facing water scarcity and requiring extra water to maintain yields. This growing mismatch between where crops are grown and where resources, especially water, are abundant highlights the need for smarter, resource‐informed crop placement and water management. Aligning crops with local environmental capacity represents an opportunity to ease pressure on finite resources, strengthen food security, protect ecosystems, and ensure long‐term economic sustainability.

## Graphical abstract

Climate change reshapes the spatial alignment between crop production and environmental resources. Using multi‐source data and a crop model, integrated climatic, water, and soil endowments for maize and wheat are quantified and compared with harvest distributions. Results reveal growing mismatches, especially with water availability, indicating opportunities for resource‐informed crop placement to reduce pressure, enhance resilience, and improve sustainability.

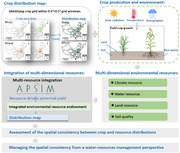

## 1 Main

Climate change is forecast to reduce yield by up to 23% in globally important staple cereal crops [1]. Many efforts, including nutrient management [2, 3], irrigation [4, 5], changes in cultivar [6, 7], and other adaptative strategies such as crop migration [8, 9], have been considered to alleviate negative impacts of climate change on crop production. As all farming systems changes have co‐benefits and trade‐offs [10, 11], some adaptations may benefit from climate change [8, 9], particularly those with low cost and integrated social, economic and environmental benefits [12, 13]. Crop migration has been considered as a plausible strategy due to benefits in dealing with both the positive and adverse impacts of climate change. For example, expanded planting of multiple cropping systems benefited from climate warming may result in ∼8 million tons of total production increase of maize, wheat, and rice in China [9], engendering future food security [14, 15]. Crop migration toward areas with reduced exposure to adverse climate conditions contributed to moderating the damaging impacts of global warming on crops and thus could avoid half of agriculture profits loss [8, 12].

However, yield benefits obtained from crop migration may come from the increment in environmental resources including more land, water, and energy use [8, 16], leading to increased environmental resources competition and risk [17]. Unfortunately, the risk of degradation of environmental resources has been intensifying over the past few decades, especially in land and water. Terrestrial ecosystems are facing a widespread regime shift from energy to water limitation [18]. Many productive land and water ecosystems have faced increasingly intensive risk and pressure during the past decades, and future agricultural production will depend upon managing the risks to land and water [19, 20, 21], with costs increasing as supply diminishes [22]. These issues call for the development of spatially‐explicit maps that highlight both key environmental resource and those pertaining to agricultural production. And these maps could guide the evaluation of sustainability in resource supply and, accordingly, facilitate insights into historical and future crop migration based on the trade‐off between environmental and crop production needs.

At present, many purposeful crop migration strategies have been proposed to address one or some of the multi‐dimensional goals of agricultural production related to environmental‐impact reductions, meeting crop demands and improving economic profits [23, 24, 25, 26, 27, 28]. These goal‐oriented frameworks always focus on the satisfaction of the total goals by observing the increase or decrease in target benefits in a large region, and are somewhat weak in resources supply perspective, leading to limitations in evaluating and improving consistency between crop distribution and current environmental resources. However, this resource‐based consistency between crop and environment may be the key to achieve sustainable crop production in the limited cropland and water resources in the future [8, 16].

China feeds 22% of the world population with only 7% of the arable land, and has become one of the world's major countries facing serious water scarcity [19, 29]. Current crop production patterns along with the extensive irrigation demand is increasing water resources scarcity in China [29, 30, 31].

Against this backdrop, there is an urgent need for integrated frameworks that jointly consider environmental resources encompassing climate, blue water and soil, when evaluating where and how crops are grown, and that move beyond single‐factor analyses of either climate or water by first integrating these resources into a common endowment framework and then disentangling their respective contributions to crop distribution. Here, we focus on China's largest dryland grain crops, maize and wheat (Figures S1 and S2), and develop a spatial environmental resource framework (see Material and Methods, Figure 6) that i) quantifies multi‐dimensional environmental resource endowments by assessing resource‐driven potential yield at 0.5° resolution, ii) diagnoses spatiotemporal mismatches between crop distributions, actual yields and environmental resource endowments, and iii) identifies water‐surplus and structurally water‐deficit regions, thereby providing a resource‐informed basis for future strategic crop distribution planning and water‐saving policies in China. Based on this framework, we conducted this study in the following phases.

**FIGURE 1:**
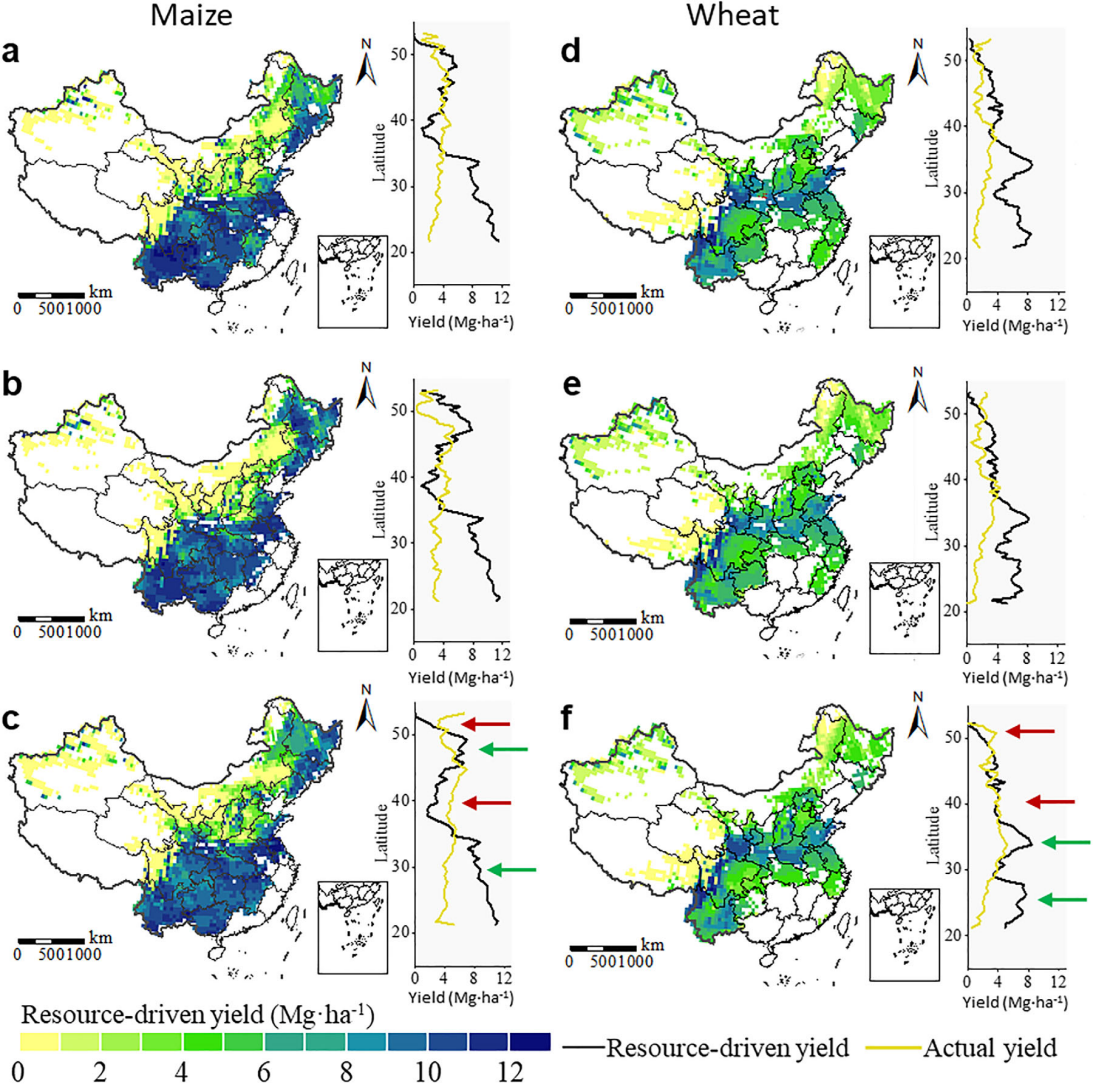
Environmental resource endowment and actual yield for maize and wheat in 2000 (a,d), 2010 (b,e) and 2020 (c,f). The maps depict geospatial distribution of climate‐water resource‐driven yield (RY) under current soil condition for each crop, among which the darker colors indicate higher values, i.e. more desirable climate‐water resources for production of each crop. The right‐side plots show the variation of RY and actual yield (AY) across latitude, with black and yellow lines depicting RY and AY, respectively. Arrows point to regions with large gaps between RY and AY. Green arrows indicate areas with high resource capacity, characterized by high RY but low AY, while red arrows present areas of resource over‐extraction, where AY exceeds RY.

First, we identified the climate‐water‐land‐soil resources supply nexus for wheat and maize. The multi‐dimensional environmental resource nexus related to climate, water, and soil in crop production was built for each 0.5 × 0.5° crop grid cell based on a bottom‐up integration of multi‐source data (See Materials and Methods section). Second, this environmental resource supply nexus was used as input to drive a crop model and quantify yield outcomes that represent local environmental resource endowment for crop production. Two crop yield levels, i.e. climate‐water resource‐driven yield (RY, Figure 1) and climate resource‐driven yield (CRY, Figure S4), were simulated to reflect climate‐water and climate resource endowments under current soil conditions, respectively, thereby developing a detailed environmental resource endowment map for these crops. These maps reflect climate and water resource capacity for crop distribution across the nation. By combining with actual yield (Figure S3), we illustrated the expansion potential for the crop in a specific region, aiming to contribute to a more adaptative and sustainable crop redistribution strategy in China. Then, we conducted an equality evaluation of environmental resource endowment to explain the consistency between crop distribution and environmental resources. A greater level of equality corresponds to higher natural endowment in large distribution areas, and on the contrary, greater inequality corresponds to higher natural endowment in a few areas of maize or wheat. Thus, the equality evaluation can indicate the consistency of distribution between crop and environmental resources. Furthermore, the surplus and deficit of resource supply were clarified. These data demonstrated the production gap between environmental resource endowment and the actual crop production, providing a reference for managing the inconsistency between crop distribution and environmental resources, and achieve agriculturally sustainable development targets.

## 2 Results

### 2.1 Special Patterns of Integrated Environmental Resource Endowment and Emerging Risk

Here, the climate‐water resource driven yield (RY) under current soil conditions was simulated at the pixel‐level to indicate the integrated environmental resource endowment for crops. It represents the maximum yield attainable under local climate, soil and blue‐water resource conditions. Actual yield (AY) denotes the realized yield under current management and socio‐economic constraints. The difference between RY and AY therefore reflects the degree to which local environmental resource endowments are under‐utilized (RY > AY, resource surplus) or nearly saturated (RY is approximately equal to AY), with AY approaching or exceeding RY indicating resource‐constrained or potentially over‐exploited production. Thus, when combined with actual crop yield (Figure S3), the gap between RY and AY provides a partial indication of regions with environmental resource surplus.

For maize, the RYs in 2000, 2010, and 2020 ranged in 0–12.81, 0–12.62, and 0–12.63 Mg ha^−1^, with means of 5.94, 6.12, and 5.96 Mg ha^−1^, respectively (Figure 1a–c). High RYs predominantly occurred in the southern and central regions. Compared with the AY, RY exhibited more significant latitudinal differences, indicating significant gaps between RY and AY in certain areas. In low‐latitude regions, higher RY than AY shows a considerable climate‐water resource endowment for maize production. While near 40 and 50°N, the positive gains in AY compared to RY have indicated the excessive water extraction in these regions. RYs of wheat in 2000, 2010, and 2020 ranged from 0–12.00, 0–11.91, 0–12.10 Mg ha^−1^, with mean values of 4.82, 4.63, 4.72 Mg ha^−1^, respectively. High RYs are concentrated primarily in the southwest and central regions. Similar to maize, the RY of wheat exhibited latitudinal differences. A high climate‐water resource endowment for wheat was found around 35°N.

### 2.2 Reserve Shift of Main Harvest Area and High Environmental Resource Endowment Region

**FIGURE 2:**
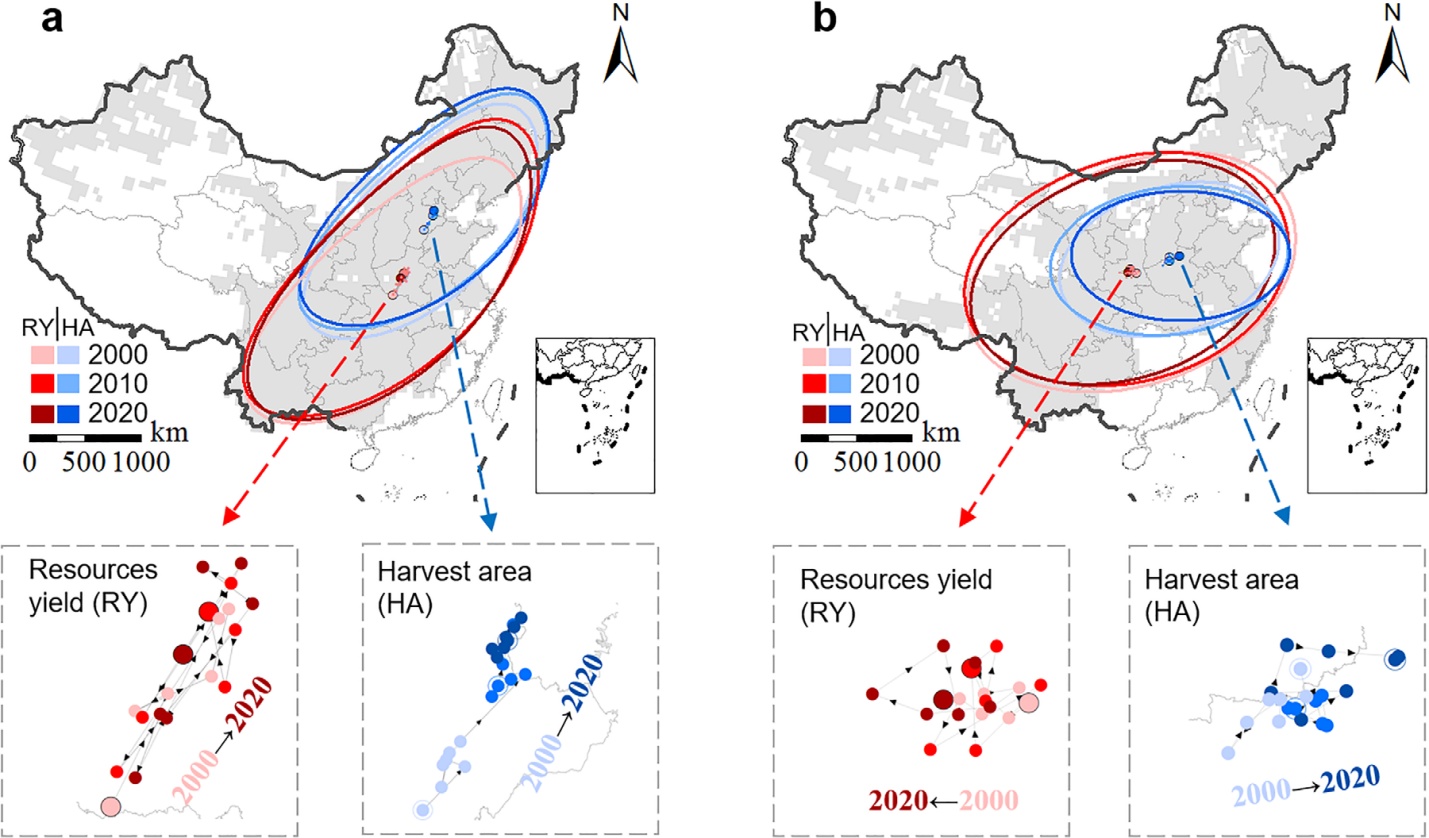
The spatial distribution patterns and temporal shifts of harvest area and environmental resource endowments for maize (a) and wheat (b). Grey blocks in the upper maps depict the distribution regions of maize and wheat during the past 20 years. Red and blue lines depict standard deviation ellipses (SDE) of climate‐water resource‐driven yield (RY) and harvest area (HA) in 2000, 2010, and 2020. Red and blue points are RY‐weighted and HA‐weighted centroids, i.e., the average location where most RY and HA are concentrated in 2000, 2010, and 2020. The lower panels illustrate the temporal shifts in RY and HA. The left sub‐panels refer to the movement trajectories of RY centroids (in red), while the right sub‐panels refer to the movement trajectories of HA centroids (in blue). The largest three points are centroids in 2000, 2010, and 2020, and the smaller points are annual centroids of other years.

Between the years 2000 and 2020, we analyzed and compared the distribution dynamics of RY and harvest area (HA) for maize and wheat in China. For maize, both RY and HA followed a northeast‐southwest distribution orientation (Figure 2a). From 2000 to 2020, RY and HA exhibited a general shift to the west and north, as indicated by the movement of the standard deviation ellipses (Figure 2a). The centroids of RY remained within Henan Province, while the centroids of HA were located further north and shifted within Hebei Province. Notably, the centroids of RY showed oscillatory shifts—first northeast from 2000 to 2010, then southwest from 2010 to 2020—while the centroids of HA consistently shifted northeast throughout the entire period. As a result, by 2020, the spatial distance between the centroids of RY and HA had increased, exposing a growing misalignment between crop distribution and resource endowment. This misalignment stemmed from the fundamental difference in determining factors. In contrast to resource endowment, which was predominantly shaped by climate, crop distribution was primarily influenced by various anthropogenic and socioeconomic drivers [32]. The observed spatial misalignment between maize migration and high‐resource‐endowment regions underscores the critical need for strategic optimization of crop distribution to enhance resource‐use efficiency, mitigate environmental stress, and promote long‐term agricultural sustainability.

For wheat, the distribution of RY followed a slight northeast‐southwest orientation, while HA exhibited a clear east‐west orientation (Figure 2b). The standard deviation ellipses of RY shifted westward, whereas those of HA shifted eastward and became smaller over the same period. The significantly smaller standard deviation ellipses for HA compared to RY suggest that the harvest areas are more concentrated, indicating a substantial geospatial resource potential for expanding wheat area. In terms of spatial location, centroids of RY shifted westward within Shaanxi Province, while the centroids of HA moved northeast, covering northern Henan and southeastern Shanxi Provinces. This contrasting directional movement of the centroids between RY and HA contributed to an increasing inconsistency between crop distribution and resource availability. These shifts underscore the growing spatial misalignment between crop harvest areas and the natural resources necessary to sustain them, particularly in relation to water resources, which may exacerbate resource stress in the coming decades.

### 2.3 Worsening Misalignment between Crop Distributions and Water Resources

We calculated the consistency between HA and RY in two scenarios: the actual scenario and the counterfactual scenario. Actual consistency was based on the current actual crop distribution and resource endowment, reflecting the degree of alignment between HA and environmental resources in the present agricultural landscape. Counterfactual consistency assumed an ideal scenario where crop areas are redistributed to perfectly align with resource availability, resulting in no inconsistency between HA and RY. This counterfactual scenario serves as a baseline, indicating the inherent inconsistency caused by the unequal distribution of resources across crop production regions. The gap in consistency between the two scenarios highlights the degree of mismatch caused by crop distribution not being synchronized with resource distribution. A larger gap signifies a more pronounced misalignment between crop production and resource endowment, emphasizing the need for strategic crop distribution to optimize resource use and ensure agricultural sustainability.

**FIGURE 3:**
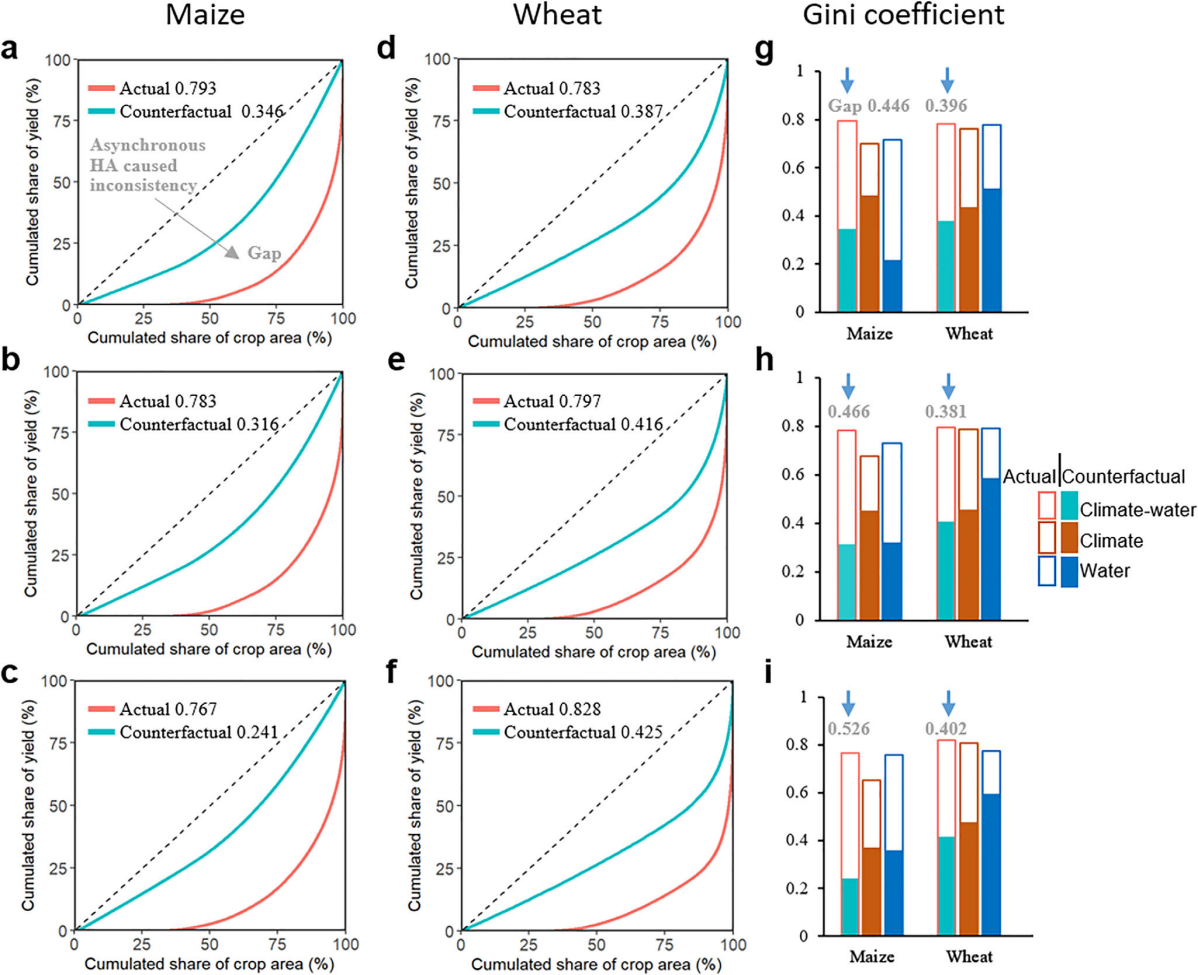
Lorenz curves and Gini coefficients for environmental resources of maize and wheat in 2000 (a, d, g), 2010 (b, e, h), and 2020 (c, f, i). The dashed lines in the Lorenz curves represent the lines of absolute consistency between the distribution of crop and resource, indicating a perfectly equal distribution of resources across all harvest areas. A greater deviation of the Lorenz curve from this line indicates higher inequality and a larger Gini coefficient. Blue Lorenz curves under the assumed counterfactual scenario, where crop areas are redistributed to perfectly align with resource availability, indicate the deviation from the absolute consistency line caused by resource disparities. Red Lorenz curves illustrate the total deviation from the line of absolute consistency, attributable to disparities in resources and the asynchronous distribution of crops relative to resource allocation. Bar charts illustrate Gini coefficients and the corresponding gaps under two scenarios.

**FIGURE 4:**
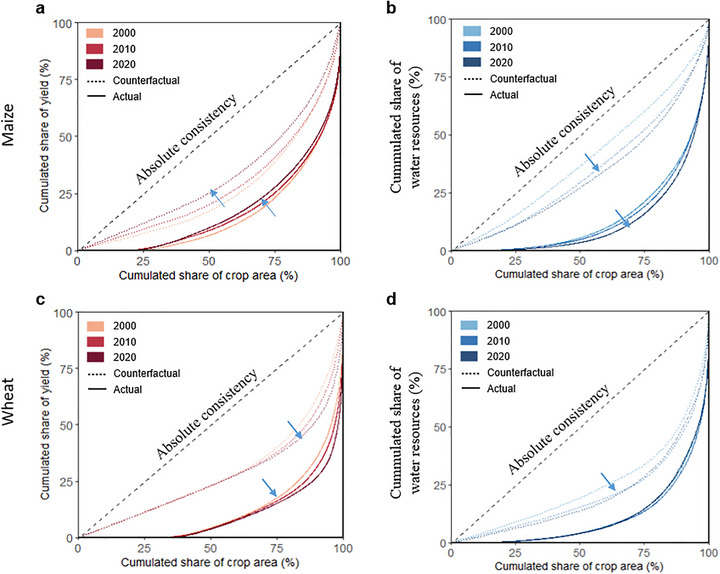
Lorenz curves for climate and water resources of maize (a, b) and wheat (c, d). The arrows denote the deviation direction of the Lorenz curves from the line of absolute consistency from 2000 to 2020. Arrows pointing toward absolute consistency line indicate reduced deviation and improved consistency, whereas those pointing away suggest increasing inconsistency.

Gini coefficients, derived from Lorenz curves, are used to quantify inconsistency between resources and HA. A larger deviation of the Lorenz curve from the absolute consistency line corresponds to a higher Gini coefficient, signifying greater inconsistency between crop distribution and resource allocation. From 2000 to 2020, Gini coefficients of RY reveal significant shifts under both actual and counterfactual scenarios (Figure 3). For maize, a slight reduction in HA‐RY inconsistency (−3.3%) was observed, with the Gini coefficient decreased from 0.793 in 2000 to 0.767 in 2020 under the actual scenario (Figure 3a–c). This reduction was driven by a 30.3% decline (Gini coefficient in counterfactual scenario decreased from 0.346 in 2000 to 0.241 in 2020) in regional resource disparities, especially in climate resources (Figure 4a). However, gaps of Gini coefficients between two scenarios increased by 17.9% (Figure 3g–i), highlighting an asynchronous crop distribution with climate‐water resource. In terms of separated resource dimensions, the 6.4% improvement in the alignment between maize HA and climate was observed (Figure 3g‐i), resulted from a 24.0% reduction in climate resource disparities from 2000 to 2020 (Figure 4a). In contrast, a markedly increase (63.5%) in water resource disparities have intensified the inconsistency between maize distribution and water resource (Figure 4b). The reduced climate disparities while exacerbated water disparities call for additional attention on water resource management to enhance the alignment between maize distribution and resource distribution, thus achieving efficient resource utilization.

For wheat, greater regional disparities in climate and water resources exacerbated the HA‐RY inconsistency (+5.7%) between 2000 and 2020 (Figure 3 and Figure 4c,d). Gini coefficients of RY under actual (counterfactual) scenarios increased from 0.783 (0.387) to 0.828 (0.425), with sustained gaps indicating asynchronous distribution (Figure 3d–i). Climate resource disparities rose by 6.0% and 9.9% under actual and counterfactual scenario, with a slight increase in the gap between two scenarios to 0.331 by 2020 (Figure 4c). Water resource disparities grew significantly (+15.3%), reflecting persistent misalignment although the gap decreased by 30.2% (Figure 4d).

These results highlight contrasting trends for maize and wheat, with maize showing reduced in climate resources but increasing water resource disparities, and wheat exhibiting worsening disparities in both climate and water resources. Addressing these mismatches through strategic crop distribution is essential for sustainable agricultural development.

### 2.4 The Potential of Water Resource Management in Mitigating Water Deficits

**FIGURE 5:**
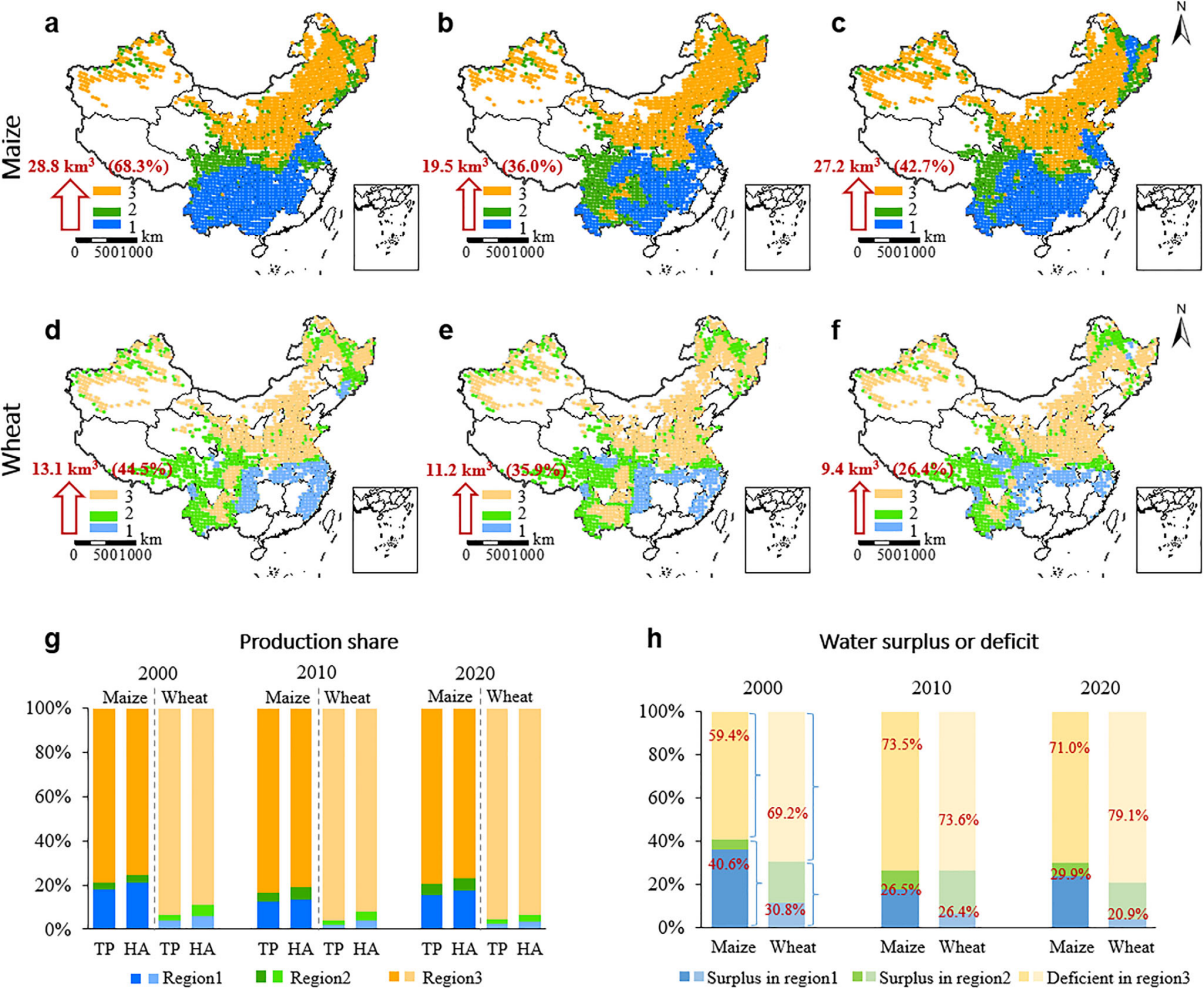
Surplus and deficient water resources for maize and wheat. Maps show water surplus and deficit regions for maize (a–c) and wheat (d–f), The red arrows, numbers, and percentages in the lower‐left corner of the map represent the potential of water reallocation to address water deficits. The numbers represent total surplus water resources in Regions 1 and 2, while the percentages show the proportion of water deficit in Region 3 that can be addressed. Plot g shows total production (TP) and harvest area (HA) shares for each region in the national total. Plot h shows surplus (for Regions 1 and 2) and deficit water share (for Region 3) in the national total water trade‐off.

Compared with climatic conditions, crop distribution exhibits a consistently stronger and increasing spatial consistency with blue water resources across both crops. Because water resources are more amenable to regulation than climate, this provides an opportunity to enhance the alignment between crop and water availability through improved water management and allocation. We therefore identified regions of water surplus and deficit to highlight priority areas to guide more targeted water management (Figure 5). Water‐deficit regions mark zones where improved water management is urgently needed, whereas water‐surplus regions point to opportunities to address crop water deficits by managing and reallocating resources. We identified the potential water surplus regions (Region 1, where effective precipitation alone is sufficient to meet crop water requirements; Region 2, where effective precipitation is insufficient but the combination of effective precipitation and local crop water supply can fully meet crop water requirements) and water‐deficient regions (Region 3, where local water resources are insufficient to fully meet crop water requirements) for maize and wheat, and evaluated the potential of reallocation to address water deficits.

For maize, Region 3, covering Northeast, North, and Northwest China, accounted for ∼75.2%–80.8% of national harvest area (HA) and ∼79.2%–83.6% of total production (TP) from 2000 to 2020 (Figure 5a‐c. Approximately 83.6% of maize production faced water deficits, requiring additional blue water input of 42.1, 54.2, and 63.7 km^3^ in 2000, 2010, and 2020, respectively. Excessive groundwater extraction for irrigation, particularly in North China, has exacerbated water scarcity [33]. Regions 1 and 2, located in South and Southwest China, offered surplus water of 25.5 (3.2), 13.1 (6.4), and 21.9 (5.3) km^3^, potentially resolving 68.3%, 36.0%, and 42.7% of deficits in Region 3 if fully utilized.

Wheat faced even greater blue water deficits. Region 3, mainly in North China, accounted for ∼89%–93.5% of HA and ∼93.6%–96% of TP, with over 90% of wheat production requiring additional water inputs of 29.4, 31.2, and 35.4 km^3^ in 2000, 2010, and 2020, respectively (Figure 5d‐f,g). Regions 1 and 2, located in Central, Southwest, and Northeast China, contributed only 6.1% and 4.9% of HA in 2000, declining to 3.4% and 3.1% by 2020. Surplus water of 5.0 (8.2), 2.7 (8.5), and 1.8 (7.6) km^3^ in Regions 1 and 2 could alleviate 44.5%, 35.9%, and 26.4% of Region 3's deficits.

It must be emphasized that the blue water deficits quantified here are derived from our annual‐scale water constraint assessment. Consequently, they represent persistent, structural shortages within Region 3 and systemic, annual surpluses in Regions 1 and 2. While this clarifies the broad spatial mismatch, the critical intra‐annual timing mismatch, where surplus and deficit periods may not coincide within a growing season, remains unresolved by our analysis. Therefore, the calculated potential for surplus reallocation represents an optimistic, theoretical upper bound.

## 3 Discussion

### 3.1 Climate‐Water Resource for Crop Production

A previous study has demonstrated widespread mismatches between the global distribution of major food crops and climate suitability [34]. Our results are broadly consistent with these studies in showing that climate gradients play an important role in determining crop suitability. However, by explicitly integrating water resource availability into a crop modeling framework, we reveal an additional and critical dimension of crop‐environmental resource mismatch that is not captured by climate‐only analyses. In China, regions that appear climatically suitable for maize and wheat production often experience substantial blue water deficits, indicating that climate suitability alone can mask underlying water stress. Moreover, whereas previous studies primarily provide static diagnostics of crop–climate mismatches, our analysis further captures the temporal evolution of crop–resource consistency over the past two decades. This dynamic perspective enables the identification of emerging water‐deficit hotspots driven by climate variability and increasing water demand, thereby offering a more process‐based understanding of climate‐water constraints on crop production under ongoing climate change.

Specifically, our analysis revealed growing regional disparities in climate‐water resource for crop production in China, particularly for maize. The RY exceeded 10 t ha^−1^ in Southwest China and Central China but was less than 2 t ha^−1^ in North China and Northwest China (Figure 1a–c), driven by pronounced water resources differences. In North China and Northwest China, high climate resource yield (CRY, representing potential yield under optimal light‐temperature conditions) was observed (Figure S4), consistent with previous studies [35, 36, 37]. These regions experienced substantial yield gaps between CRY and RY, underscoring water resources limitations as a major constraint on productivity (Figure S5). Moreover, actual yield (AY) in these areas exceeded RY, suggesting over‐extraction of environmental water flows to sustain high yields, exacerbating groundwater depletion [38]. High resource endowments for maize were in Northeast China and Southern China, and for wheat in Central China (Figure 1), indicating substantial production potential in these regions. The observed regional disparities in resource endowment highlight opportunities to align crop distribution with resource availability, balancing production demands with environmental sustainability.

### 3.2 Managing Crop‐Resource Distribution Inconsistencies

We observed longitudinal improvement in inconsistency between HA and RY for maize but worsening inconsistency for wheat (Figure 3). For maize, HA and RY centroids shifted northeastward, while for wheat, HA centroids moved eastward, diverging from westward‐moving RY centroids (Figure 2). These patterns suggested that climate warming has extended growth seasons and reduced chilling risks in Northeast China and North China [39, 40], enhancing CRY in these regions [9, 39]. These increased spatiotemporal distribution potential under changing climate, coupled with other global and local anthropogenic, biophysical, social‐economic, and policy drivers, including cropping structure change under rapid urbanization and the continuous loss of rural labor, frequent climatic disasters, and large areas of abandoned farmland in southern and southwestern China [32, 41], catalyzed the continual northward movement for maize and continually concentrating in the main production areas of North China Plain for wheat production. However, the resulting crop distribution misaligned with regions of high environmental resources, particularly water, leading to substantial inconsistencies (Figure 4). It is plausible that cropping patterns eventually follow environmental resources, with cropping areas appearing and disappearing over time following water, energy and other natural resource availability or depletion, but such trends would also be underpinned by social, economic, practical and policy factors [42, 43].

Our findings highlight the Northern China Plain as a hotspot for resource mismatch, with HA concentrated in areas facing declining water resources [44], whereas the centroids of RY observed in water‐abundant southern regions. These patterns suggest opportunities to better align crop distribution with regional resource capacities. Particularly, reducing crop area in resource‐deficient regions such as North and Northwest China, and cautiously increasing production in resource‐abundant areas, could help relieve water stress while sustaining grain output. It should be noted that many of resource‐abundant regions identified are also major production bases for rice or other staple crops. Although our calculation of environmental resource endowments already accounts for the water use of other crops, any practical adjustment of maize and wheat distribution would still need to be weighed against existing local cropping patterns and production priorities. Future work that jointly evaluates multiple major crops could provide a more comprehensive basis for designing coordinated, system‐wide solutions. Such changes would require a combination of both top‐down and bottom‐up interventions, including policy and regulatory instruments as well as participatory engagement with local farmers [45, 46].

Additionally, water resource management offers potential solutions. Physical water transfers, though energy‐intensive and costly [47], could help redistribute water to low RY but high CRY regions like Northern China, reducing reliance on groundwater and mitigating environmental degradation [29, 48]. We evaluated the excess water demand in regions with resource deficits (Figure 5) to provide a reference for policy interventions aimed at sustainable resource allocation. The spatial pattern of water deficits and surpluses presented here is a function of our annual‐scale analytical framework. This perspective effectively highlights long‐term, regional‐level water insecurity in Region 3 and potential macro‐scale water sources in Regions 2 and 1. However, precisely because our maps cannot identify acute, growing‐season‐specific shortfalls, any policy strategy relying on inter‐regional water allocation must be intricately coupled with investments in local, in‐season water management (e.g., storage, scheduling) to address the inevitable timing gaps. Furthermore, our analysis employs a uniform irrigation efficiency factor and does not account for substantial regional differences in irrigation methods, infrastructure, and management. In systems like the flood‐irrigated fields, low field application efficiency means the actual volume of water that must be abstracted from sources is significantly higher than our net deficit estimates suggest. Therefore, any strategy for reallocating water resources must be designed and implemented in tandem with major investments in modernizing irrigation infrastructure (e.g., transitioning to drip or sprinkler systems) and improving water management practices. Our estimates should thus be viewed as highlighting regions where the joint benefits of efficiency gains and reallocation could be greatest.

### 3.3 Limitation and Future Recommendations

Our study used simulated yields under the climate‐water‐soil nexus to represent resource endowment, offering a comprehensive approach compared to single‐dimension indicators like radiation or water availability. It is important to note that this analysis was conducted at a 0.5° (approximately 50 km) grid resolution. While suitable for identifying broad regional patterns and contrasts, consistent with recent large‐scale crop‐climate assessments [49], this resolution cannot fully capture fine‐scale heterogeneity in climate, soil, and management practices, particularly in smallholder systems. Thus, the results serve as regional scale indicators rather than field or farm level decision support. This framework identifies broad spatial patterns and relative contrasts in environmental resource endowments and climate‐water constraints, helping to prioritize regions for adaptation and water management interventions. However, several limitations persist: 1) irrigation efficiency and management: regional variations in irrigation efficiency and practices were not incorporated, potentially underestimating yields in well‐irrigated regions like Northern China. Future studies should integrate detailed data on irrigation schedules, infrastructure, methods, and efficiencies to refine yield estimates under current environmental conditions [46, 50, 51]. 2) water resource function: the distinct roles of rainfall and irrigation were not separately analyzed, limiting the development of tailored strategies for addressing water shortages from these sources. 3) seasonal variations: a core limitation of our water scarcity analysis stems from the use of annual water budgets. This approach does not capture sub‐annual variability and timing mismatches between water supply and critical crop growth stages. Consequently, our model likely underestimates the severity of episodic water stress and production risk in regions where rainfall is poorly distributed seasonally, even if annual totals appear adequate. Addressing temporal inconsistencies in water resource availability should be a focus of future research, to better align crop production with dynamic water patterns. Furthermore, incorporating higher‐resolution soil, management, and remote‐sensing data would help represent sub‐grid heterogeneity and refine resource‐informed adaptation strategies. By addressing these limitations and leveraging more detailed regional data, future studies can offer actionable insights for sustainable crop distribution and resource management.

## 4 Materials and Methods

### 4.1 Data Source

**FIGURE 6:**
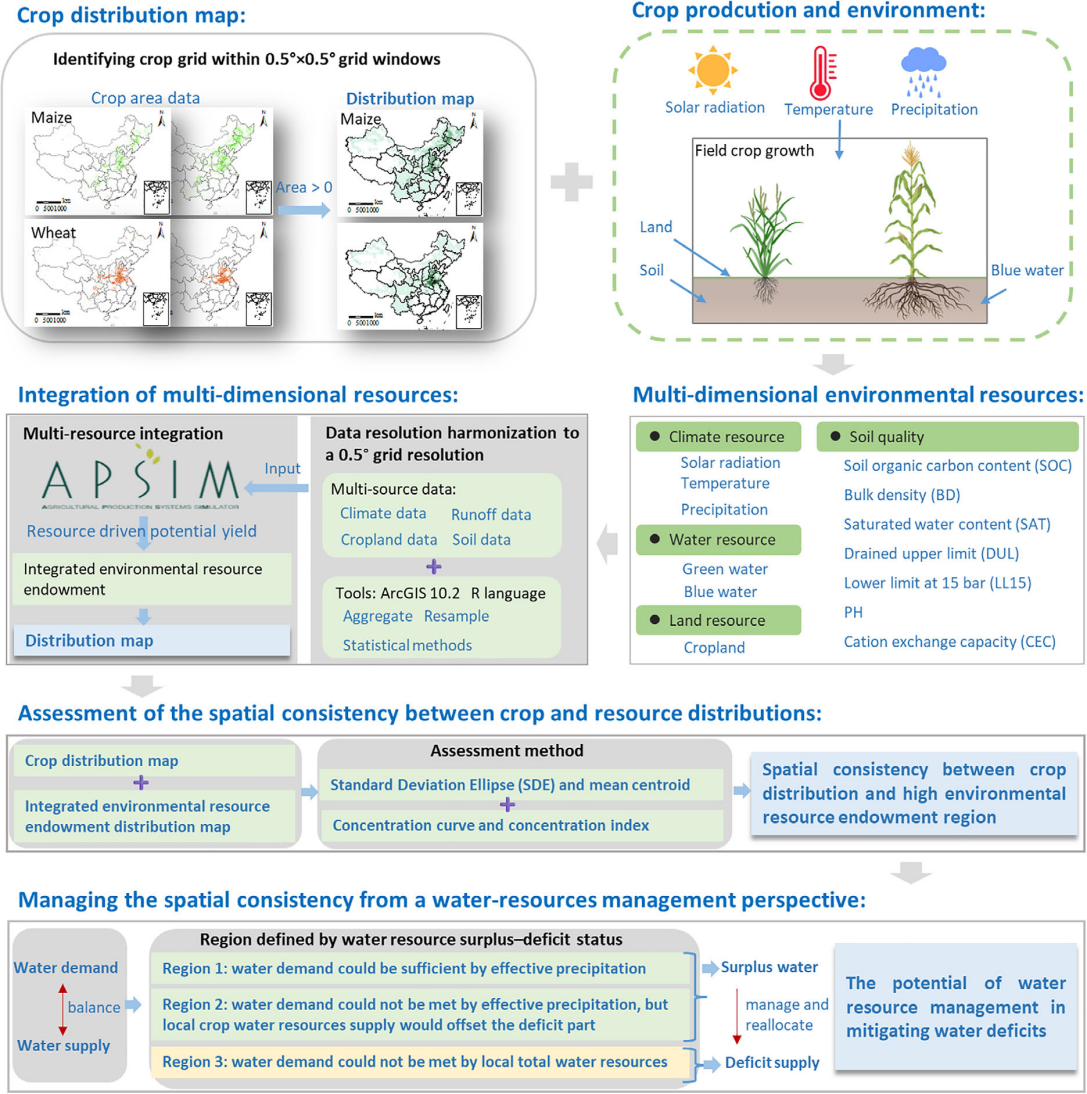
Framework for integrated quantitative assessment of multi‐dimensional environmental resources and crop–resource spatial consistency. Crop distribution maps for maize and wheat are first derived from crop area statistics at a 0.5° grid resolution. Multi‐source climate, runoff, cropland and soil datasets are then harmonized to the same grid and, together with APSIM simulations, used to estimate resource‐driven potential yield and construct an integrated environmental resource endowment that combines climate, water (green and blue), land and soil‐quality attributes. Spatial consistency between crop distribution and environmental resource endowment is quantified using standard deviational ellipses and mean centroids, as well as concentration curves and a concentration index. Finally, regions are classified according to the balance between crop water demand and local water supply (effective precipitation and local water resources), thereby identifying water‐surplus and water‐deficit areas and assessing the potential of water‐resources management and reallocation to mitigate crop water deficits.

Given crop growth dependence on natural resources such as radiation, heat, water, and land, we considered a multi‐dimensional environmental resource nexus based on climate, water, and soil to assess the resource endowment for crop growth (Figure 6). The multi‐dimensional environmental resource nexus was built on bottom‐up multi‐source data integration. First, the 1 km × 1 km resolution annual harvesting area of maize and wheat was used to identify the detailed distribution of these two crops, and was derived from the National Ecosystem Science Data Center, National Science & Technology Infrastructure of China (http://www.nesdc.org.cn). A 30 m spatial resolution cropland dataset was used to identify the land chance for each crop [52]. For climate conditions, the gridded spatial climate data at 0.5° (approximately 50 km) horizontal and daily temporal resolution were used for the characterization of climate conditions across time and space. The gridded climate data were obtained from the W5E5 v2.0 dataset, which provides bias‐corrected daily climate variables at 0.5° resolution globally, based on ERA5 and observational datasets [53]. Local total runoff including surface and subsurface, used for the calculation of available water resources, was extracted from the ERA5‐Land monthly averaged data at 0.1° (approximately 10 km) spatial resolution [54], and was downloaded from Copernicus Climate Change Service Climate Data Store (https://cds.climate.copernicus.eu). Soil data was obtained from the China Soil Scientific Database [55], which was generated based on more than 7,000 soil profile measurements across China and is the most comprehensive soil data available, downloaded from the National Tibetan Plateau/Third Pole Environment Data Center (http://data.tpdc.ac.cn). Major soil properties including SOC content, total nitrogen content, bulk density, clay fraction, pH, saturated water content, and drained upper limit and lower limit of crop water extraction in different soil layers down to 2.5 m were used to indicate the soil quality and drive the crop model. All datasets were harmonized to a 0.5° resolution using the Resample and Aggregate tools in ArcGIS 10.2 and R language. Gridded maize and wheat yield data at 0.083° (approximately 8 km) resolution were extracted from the SPAM database, including Version 3.0.7 for year 2000, Version 2.0 for year 2010, and Version 1.0 for year 2020 (https://mapspam.info). To ensure scale consistency, SPAM yields were not directly averaged across grids. Instead, yields were converted to production at the 5 arc‐minute scale, aggregated within each 0.5° grid cell, and recalculated as area‐weighted yields.

### 4.2 Observation of Maize and Wheat Distribution

We observed total area (TA) within each 0.5 × 0.5° pixel based on 1 km × 1 km annual harvested areas datasets. The harvest share in each 0.5 × 0.5° pixel (2500 km^2^) was calculated as harvest area (HA) for maize and wheat to obtain the detailed maize and wheat distribution for constructing the multi‐dimensional environmental resource supply nexus. Given the varying cropland availability across the nation, we calculated the harvest share in cropland as the crop area for maize and wheat in equality analysis to incorporate the impact of land resource. Total cropland in each pixel was observed based on the 30 m spatial resolution cropland dataset.

### 4.3 Quantification of the Environmental Resource Endowment

The Agricultural Production Systems sIMulator (APSIM) contains a suite of modules that enable the simulation of agricultural systems for a diverse range of plant, animal, soil, climate, and management interactions and is internationally recognized as a highly advanced platform for modeling and simulation of agricultural systems (https://www.apsim.info/). Currently, APSIM has been widely used in the simulation of climate‐plant‐soil interactions and the impact of management intervention [56, 57, 58, 59, 60]. Here, APSIM is considered as a useful tool for integrating multidimensional resources and used to simulate crop growth under the climate–water–soil nexus on a daily time step after being well‐validated in our study region in previous studies (See Supporting Method 1).

Using the well‐validated APSIM 7.10, we simulated crop yields at two production levels to represent the environmental resource endowments for crop production. We first simulated a growth level that is explicitly constrained by local climate, available blue water resources, and soil conditions. The resulting yield presents the maximum achievable under these local environmental resource constraints—solar radiation, temperature, precipitation, soil properties, and limited irrigation based on local blue water supply. We defined this as the climate‐water resource‐driven yield (RY) under current soil condition, which serves as an indicator of integrated environmental resource endowment across regions.

In regional field production, climate resources, especially solar radiation and temperature, are difficult to manipulate, and their combined effects largely determine the upper limit of crop productivity across regions [61, 62]. To isolate the respective roles of climate, here represented by the coupled radiation–temperature regime, we simulated a second production level, defined as crop production primarily constrained by these two factors under current soil conditions. The yield corresponding to this level, hereafter referred to as the climate resource‐driven yield (CRY), was used as an indicator of the climate resource endowment for crop production. Together, RY and CRY provide two complementary indicators that quantify integrated environmental climate resource endowment for maize and wheat across regions.

Using the assimilated multi‐dimensional environmental resource datasets harmonized to a 0.5° grid, we simulated RY and CRY at a daily time step from sowing to maturity for all identified crop grid cells for each year during 2000–2019 (Figure 6). For the simulation of RY at each grid cell, daily climate data and layered soil data were input into model to reflect climate condition (solar radiation, temperature, and precipitation) and soil properties. Blue water resources were used to supply irrigation for crop growth. Crop water supply (CWS) represents the total annual volume of blue water available for irrigating a given crop. In the simulations, this water source was applied through an automatic irrigation module triggered by soil moisture conditions (see Supporting Method 1).

Regarding crop management, we selected representative cultivars that are widely grown in actual production for each province. Sowing dates were set based on publicly available gridded datasets developed from ground‐based observations and remote sensing data. We assume that crops were grown under non‐nitrogen‐limited conditions to assess the maximum yield achievable under current environmental resource conditions. When simulating CRY, we applied the same settings as for RY, except that irrigation constrained by CWS was replaced with full irrigation, so that crop growth was no longer limited by water availability and was primarily constrained by the climatic regime.

### 4.4 Calculation of Water Requirements, Water Supply, and Water Availability for Crop

We assessed the water resources based on crop water availability (CWA), determined by the water demand and supply for crops. Total crop water requirements (CWR) for maize and wheat were calculated first. Given two different water sources for crop production from rainfall and irrigation, we calculated both green (GWR) and blue water requirements (BWR). Where, green water refers to the effective precipitation consumed during the growing period of a crop. Blue water refers to the amount of water that needs to be supplemented by irrigation when effective precipitation during the crop‐growing season is insufficient to maintain normal crop growth. CWR, GWR, and BWR were calculated using a process‐based crop water model [27]. Detailed calculation can be seen in Supporting Method 2.

Crop water supply (CWS) is referring to water resources that could be extracted to support crop irrigation manually when crop water requirement could not be meet by local effective precipitation. Crop water supply was decided by the remaining part of total available water resources excluding environmental flow requirements and other uses of industrial and domestic water consumption. Given to higher priority for water use in other human water use sectors (OUs) including industrial, urban, and rural sectors, we calculated the remaining water excluding OUs as available water supply (AWS, which we defined as water resource endowment) for all crop production. To eliminate the influence of water resource competition with other crops, we further deducted the irrigation consumption of other crops including rice, vegetables, and other cultivated plants based on their water use shares in total irrigation water consumption. Total available water supply (TWS) for maize and wheat within each pixel could be determined. Combining the crop harvest area, detailed crop water supply (CWS) for maize and wheat were calculated and could be input as irrigation in APSIM (see Supporting methods). Specific calculations followed Equations ((1), (2), (3)).

(1) $$AWS=\left(R-EF)\times (1-OU_{share}\right)$$

(2) $$TWS=AWS\times \left(1-OC_{share}\right)$$

(3) $$\begin{array}{ccc} & & CWS_{maize/wheat}\\ & & \;=TWS\times \frac{Area_{maize/wheat}\times CWR_{maize/wheat}}{Area_{maize}\times CWR_{maize}+Area_{wheat}\times CWR_{wheat}} \end{array}$$

where, R is the pixel‐level total water resources from annual runoff (mm). EF is environmental flow requirements (mm), setting as 80% of total runoff without affecting the integrity of downstream water‐dependent ecosystems and livelihoods based on literature [21]. *OU_share_* is water use share of other human water use sectors including industrial, urban, and rural sectors in total human water use. *OC_share_* is share of other crop water use in total irrigation water use. We calculated averaged *OU_share_* and *OC_share_* since 2000 based on human water use dataset in the prefecture‐level conducted by Zhou et al. [31], and downscaled to grid resolution. *CWS*_*maize*/*wheat*_ was crop water supply for maize or wheat within each pixel (mm). *Area*_*maize*/*wheat*_ was total harvest area of maize or wheat within each pixel (km^2^).

Finally, annual crop water availability (CWA) was calculated using equation (4). Larger CWA reflected relatively more water resources supply and higher water endowment. In the simulation of climate‐water resource endowment, CWA combined with CWS were input into APSIM to simulate different water resource conditions for crop production (see Supporting method 1).

(4) $$CWA=\frac{CWS}{CWR}$$

To clarify the regional water resources surplus and deficit, we characterized crop pixels into three categories based on water demand and supply: 1) Region 3, CWR> CWS + CEP, water demand could not be met by local crop water supply and effective precipitation during the crop growth period (CEP). In these regions, special attention should be given to water‐related issues in crop production. 2) Region 2, CWR ≤ CWS + CEP but CWR > CEP, water demand could not be met by effective precipitation during the crop growth period, but local crop water resources supply would offset the deficit part. 3) Region 1, CWR ≤ CEP, water demand could be sufficient with effective precipitation during the crop growth period. The deficit part of CWR in Region 3 and the surplus part of CWS in Region 2 and Region 1 for each crop were calculated based on the total area of crops (equations ((2), (3), (4))). Therefore,

(5) $$Water\;Deficit_{Region3}=\sum \left(CWR-CWS-CEP\right)\times 10^{-6}\times Area$$

(6)

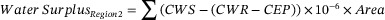



(7)

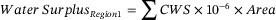

where, Water Deficit_Region 3_ was total deficit water in all pixels characterized to Region 3 (km^3^). Water Deficit_Region 2_ and Water Deficit_Region 1_ were characterized by total surplus water in all pixels characterized to Region 2 and 1, respectively (km^3^). CWR, CWS, and CEP was total crop water requirement, crop water supply and effective precipitation within each pixel (mm). Area was the total area of maize or wheat within each pixel (km^2^). CEP was calculated based on daily effective precipitation (P_eff_), which was in turn estimated from daily precipitation (P) using equation (8) according to Yin et al. [63].

(8) $$P_{eff}=\left\{\begin{array}{cc} 0 & if\;\;\;P\leq 5\;mm\\ 0.9\times P & if\;\;5\;mm<P\leq 50\;mm\\ 0.75\times P & if\;P>50\;mm \end{array}\right.$$

(9) $$CEP=\sum P_{eff}$$

### 4.5 Distribution of HA, RY, and Spatial Consistency Analysis

Standard Deviation Ellipse (SDE) and centroid were used to indicate the spatiotemporal distribution pattern of HA and RY of maize and wheat. SDE, a statistical method often used in geographic and spatial analyses to assess the extent, direction, and concentration of features or phenomena in the given area, was used to visualize the spatial distribution of crop pixels concerning their average location, while also considering the variability (spread) and orientation of the data. HA‐weighted and RY‐weighted SDEs were conducted using the Standard Deviational Ellipse tool in ArcGIS 2.0. The trajectory of HA and RY migration during 2000–2020 was further depicted by calculating the HA‐weighted and RY‐weighted centroids based on equations (10) and (11).

(10) $$X_{t}=\frac{\sum_{i=1}^{n}\left(W_{i,t}\times X_{i}\right)}{\sum_{i=1}^{n}\left(W_{i,t}\right)}$$

(11) $$Y_{t}=\frac{\sum_{i=1}^{n}\left(W_{i,t}\times Y_{i}\right)}{\sum_{i=1}^{n}\left(W_{i,t}\right)}$$

where, *X_t_* and *Y_t_* is the longitude and latitude of the centroid of the HA (RY) in China respectively. *X_i_* and *Y_i_* is the longitude and latitude of grid *i*, respectively. *W*_*i*,*t*_ is HA (RY) of the crop for year t in grid *i*. The crop is maize or wheat in this study. We calculated and visualized HA‐weighted and RY‐weighted centroids using the Mean Center tool in ArcGIS 10.2.

We estimated the consistency between crop distribution and resource endowment by introducing the concentration curve and concentration index. The ideal crop distribution should align with the distribution of resource endowment to minimize impact on environmental resources and maximize sustainable crop production, and thus reflect an absolute equality in resource endowment. We calculated the inequality in resource endowment to indicate the inconsistency between the two elements. Lorenz curve and Gini coefficient, which are widely used to measure inequality in socioeconomics and are increasingly applied to analyses related to natural sciences [17, 64, 65], were introduced to present the concentration curve and concentration index here. In this adapted context, all grid cells are ranked from lowest to highest based on their resource endowment per unit area (e.g., RY, CRY, and blue water). The cumulative share of total resource endowment is then plotted against the cumulative share of the total HA, following this ranked order. A curve along the 45° line (Absolute consistency) indicates ideal alignment, where crop area is proportional to resource endowment. A curve that deviates below this line indicates a spatial mismatch, where cropping is concentrated in resource‐poorer areas. The Gini coefficient, ranging from 0 (perfect alignment) to 1 (maximum mismatch), provides a single, comparable metric to summarize the degree of this divergence.

Given that natural resources inherently exhibit spatial heterogeneity, we constructed a counterfactual crop distribution scenario for consistency assessment. In the counterfactual scenario, the observed HA were allocated across all crop grids sequentially according to their resource endowment levels. Under this hypothetical scenario, crop distribution aligns perfectly with the distribution of resource endowment, that is, crop grids with higher resource endowment are allocated greater HA, while those with lower endowment are assigned less. This approach eliminates inconsistency caused by inappropriate crop distribution, thereby reflecting inequality solely attributable to differences in regional resource endowment and highlighting the spatial variation in resources. It should be noted that this represents an unattainable hypothetical scenario, as the reallocation of observed HA is based solely on resource endowment levels, without accounting for other constraints such as the existing cropland area within each grid cell. The actual crop distribution scenario reflects the observed HA across all crop grids. We conducted the Lorenz curve and calculated Gini in two scenarios of actual and counterfactual crop distribution, and calculated the Gini difference (the actual minus the counterfactual) between the two scenarios to indicate the actual consistency between HA and resource endowment, regional differences in resource endowment, and the inconsistency caused by inappropriate crop distribution, respectively. The Gini coefficient was calculated based on equation (12).

(12) $$Gini=1-2\int_{0}^{1}L_{\omega }\left(p\right)dp=1-\sum_{i=0}^{n-1}\left(X_{i+1}-X_{i}\right)\left(Y_{i+1}+Y_{i}\right)$$

where, *L*_ω_(*p*) is the Lorenz curve; n denotes the crop grid amount; *i* is the order of resources endowment per HA rank; *Y_i_* is the cumulated share of resources endowment of the top *i* HA; *X_i_* denotes the cumulated share of HA of the top *i* HA. Absolute equality is represented by Gini  =  0. This indicates an equal distribution of resource endowments among all HAs, and a perfect alignment between endowments and HA distribution. A value closer to 1 indicates greater inconsistency in resource allocation within HAs.

## Author Contributions

Conceptualization: **J.Z**., **C.Z**., **Z.J.L**., and **X.G.Y**. Data curation: **Z.E.S**. and **Z.T.Z**. Formal analysis: **Z.E.S**. Funding acquisition: **J.Z**. and **X.G.Y**. Investigation: **Z.E.S**. Methodology: **Z.E.S**, **J.Z**., and **X.L**. Resources: **J.Z**. and **X.G.Y**. Project administration: **J.Z**., **X.L**. and **X.G.Y**. Software: **Z.E.S** and **J.Z**. Supervision: **J.Z**. Validation: **Z.E.S** and **J.Z**. Visualization: **Z.E.S**. Writing – original draft: **Z.E.S** and **J.Z**. Writing – review and editing: **M.H.Z**., **J.S**., **B.L**., **K.L**., **J.W.M.P**., **M.T.H**., and **D.L.C**.

## Funding

This work was supported by the National Key R&D Program of China (2023YFD1500200), the National Natural Science of Foundation of China (42475199; 42205192; 42271276), the Norwegian Ministry of Foreign Affairs (CHN‐2152, 22/0013 SINOGRAIN III), the 2115 Talent Development Program of China Agricultural University, and the Youth Innovation Team of China Meteorological Administration (CMA2023QN15).

## Conflicts of Interest

The authors declare no conflicts of interest.

## Supporting information




**Supporting File**: advs74609‐sup‐0001‐SuppMat.docx.

## Data Availability

The data that support the findings of this study are available on request from the corresponding author. The data are not publicly available due to privacy or ethical restrictions.
