# Supplementary material for: An Increasing Misalignment Between Crop Distribution and Environmental Resources Under Climate Change in China
Source: Adv Sci (Weinh). 2026 Mar 2;13(27):e16290. doi: 10.1002/advs.202516290 (PMC13170205; doi:10.1002/advs.202516290)
Supplement: Supplementary file 1 — Supporting File: advs74609‐sup‐0001‐SuppMat.docx. [file ADVS-13-e16290-s001.docx]

Supplementary Materials for

**An increasing misalignment between crop distribution and environmental resources under climate change in China**

Zheng’e Su *et al.*

*Corresponding author. Email: jinzhao@cau.edu.cn (Jin Zhao), liang.xia@unimelb.edu.au (Xia Liang)

**This file includes:**

Supplementary Text

Figs. S1 to S8

Tables S1 to S3

References

Supplementary Text

**Supporting Method 1: Simulation of the environmental resource-driven yield (RY) and climate resource-driven yield (CRY)**

**Calibration and evaluation of APSIM**

Comprehensively considering environmental resources including radiation, temperature, water, and soil related to crop growth and development, we quantified yield output driven by local environmental condition as an integrated indicator of resource endowment for crops production. APSIM was widely used as an effective tool to simulate climate-plant-soil interactions and the impact of management intervention. Given our prior validation of APSIM for simulating the growth and yield of maize and wheat across the study region, as evidenced by our previously published peer-reviewed studies (Table S1), we used the well-calibrated model to simulate crop growth under multi-dimensional environmental resources in this study.

**Detailed Simulation of RY and CRY**

The RYs were simulated under local radiation-temperature-water and soil condition to present the integrated environmental resource endowment. The CRY was simulated under local radiation-temperature and soil condition to present the climate resource endowment. Here, water resources included not only green water from precipitation but also blue water from runoff (Figure 6). Therefore, RY represent a comprehensive evaluation of environmental resources supply for crop production (Table S2).

During our gridded simulation of CRY, the gridded daily climate data and layered soil data were input into model to reflect climate and soil condition of crop growth in different regions. As for the management, we set the cultivars, sowing date and density based on actual production in different regions, and full supply fertilizer to eliminate the yield limitation from these non-environmental resource factors. To separate water resources from climate, we set full irrigation to eliminate water limitation in all region and thus reflect the yield output under regional radiation-temperature. We apply the same setup except for the settings related to water resources for the simulation of RY. The water resources data were input based on Automatic Irrigation Module in APSIM. The CWS and CWA (See Materials and Methods) were input into model to reflect the water resource condition. For the regions with CWA > 1, CWS could support total crop water demand in production, full irrigation was set to reflect well water resource condition. For the regions with CWA ≤ 1, CWS could not meet the crop water demand, constrained irrigation was set. Detail set of irrigation for different water sources condition in simulation of RY could be found in Table S3.

**Supporting method 2: Calculation of crop water requirements and water resource supply for maize and wheat**

**Crop water requirement**

Firstly, daily crop water requirements (ET_c_) of maize and wheat were calculated based on the crop coefficient method recommended by the FAO (Allen et al., 1998), which was widely used in the calculation of crop water requirements (Equations S1-S3). Here, the gridded reference evapotranspiration (${ET}_{0}$) dataset at a high spatial resolution of 1 km, conducted by Peng (2024), was obtained from the National Tibetan Plateau/Third Pole Environment Data Center (<http://data.tpdc.ac.cn>). Combining the daily historical meteorological data from climate stations in China (http://data.cma.cn), we calibrated the crop coefficients ($K_{c}$) for maize and wheat in different regions (Equation S2), and then ${ET}_{c}$ were calculated based on Equation (S3). After that, the total, green, and blue water requirements of each crop were calculated, respectively (Equations S4-S7).

${ET}_{0}=\frac{0.408\triangle\left( R_{n}-G \right)+\gamma\frac{900}{T_{mean}+273}u_{2}(e_{s}-e_{a})}{\triangle+\gamma(1+0.34u_{2})}$ (S1)

where, $R_{n}$ is the net radiation at the crop surface (MJ·m^-2^·day^-1^), G is the soil heat flux density (MJ·m^-2^·day^-1^), $T_{mean}$ is the daily average temperature (°C), $u_{2}$ is the wind speed at 2 m height (m·s^-1^), $e_{s}$ is the saturation vapor pressure (kPa), $e_{a}$is the actual vapour pressure (kPa), Δ is the slope of the vapor pressure–temperature curve (kPa·℃^-1^), and γ is the psychrometric constant (kPa·℃^-1^).

$K_{c}=K_{c(tab)}+[0.04\left( u_{2}-2 \right)-0.004\left( {RH}_{min}-45 \right)]({h/3)}^{0.3}$ (S2)

where, $K_{c(tab)}$ is the crop coefficient under the standard conditions at different growth stages, based on Allen et al. (1998), ${RH}_{min}$ is the average value of the daily minimum relative humidity during a particular growth stage (%) and h is the average height of the crop during a particular growth stage (m).

${ET}_{c}=K_{c}{ET}_{0}$ (S3)

where, ${ET}_{c}$ is daily crop water requirements for maize or wheat (mm), $K_{c}$ the crop coefficients in different growth stages, ${ET}_{0}$ is daily reference evapotranspiration (mm).

$CWR=\sum{ET}_{c}$ (S4)

$GWR=\sum min({ET}_{c},P_{eff})$ (S5)

$BWR=\sum max(0,{ET}_{c}-P_{eff})$ (S6)

where, CWR, GWR, and BWR is the total, green, and blue water requirements during growth period of each crop (mm), $P_{eff}$ is daily effective precipitation (mm), was estimated based on daily precipitation (P) using Equation (S7) (Yin et al., 2016).

$P_{eff}=\left\{ \begin{aligned} \begin{matrix} 0 & if P\leq5 mm \end{matrix} \\ \begin{matrix} 0.9\times P & if 5 mm<P\leq50 mm \end{matrix} \\ \begin{matrix} 0.75\times P & if P>50 mm \end{matrix} \end{aligned} \right.$ (S7)

Supplementary Figures


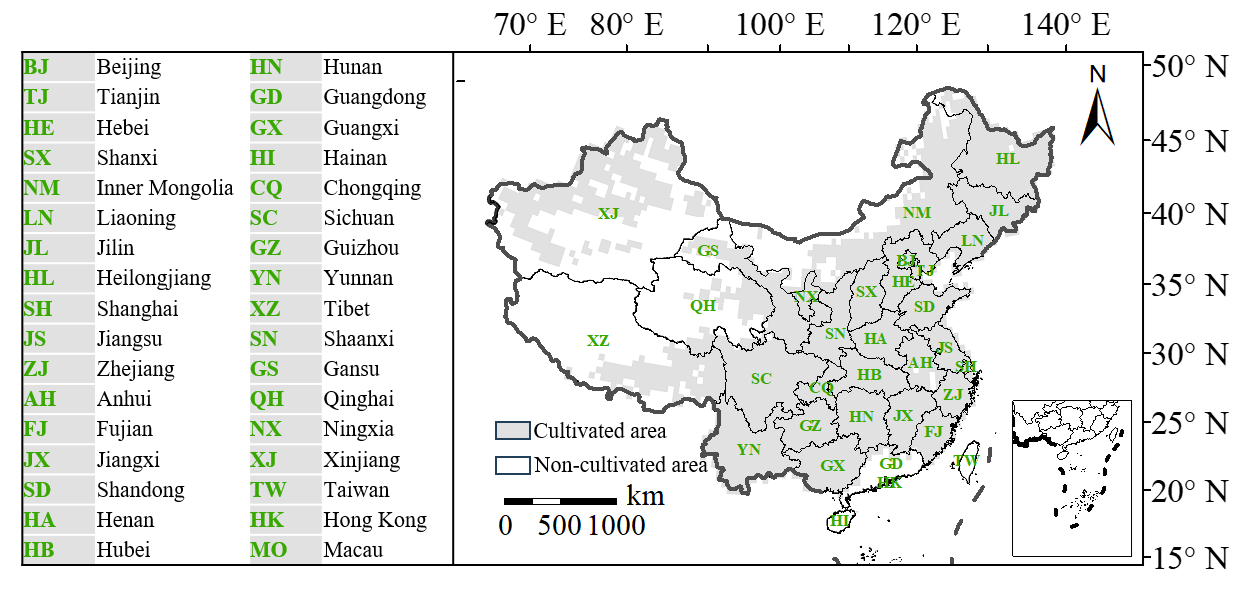


**Figure S1. Location of study region.**

The map indicates cultivated region of maize and wheat in China during 2000-2020. Green letters indicate the abbreviations of each province in China.


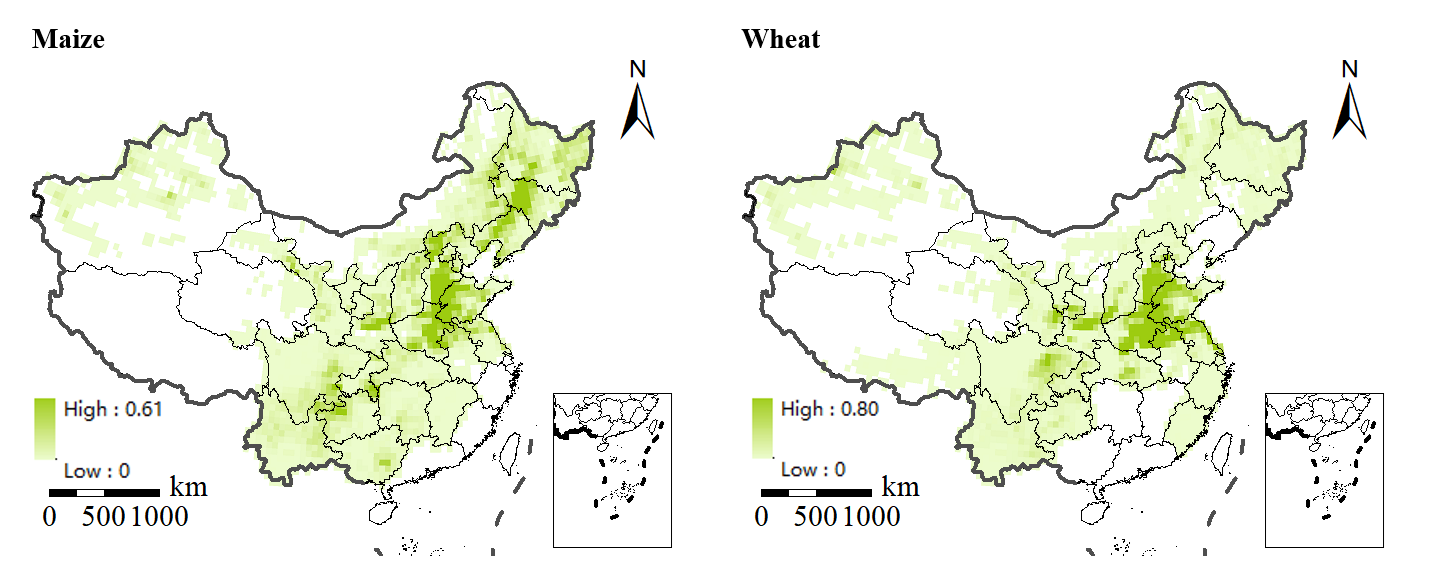


**Figure S2. Maize and wheat distribution in China.**

Maps show averaged harvest share of maize and wheat in each distributed pixels in China during 2000-2020.


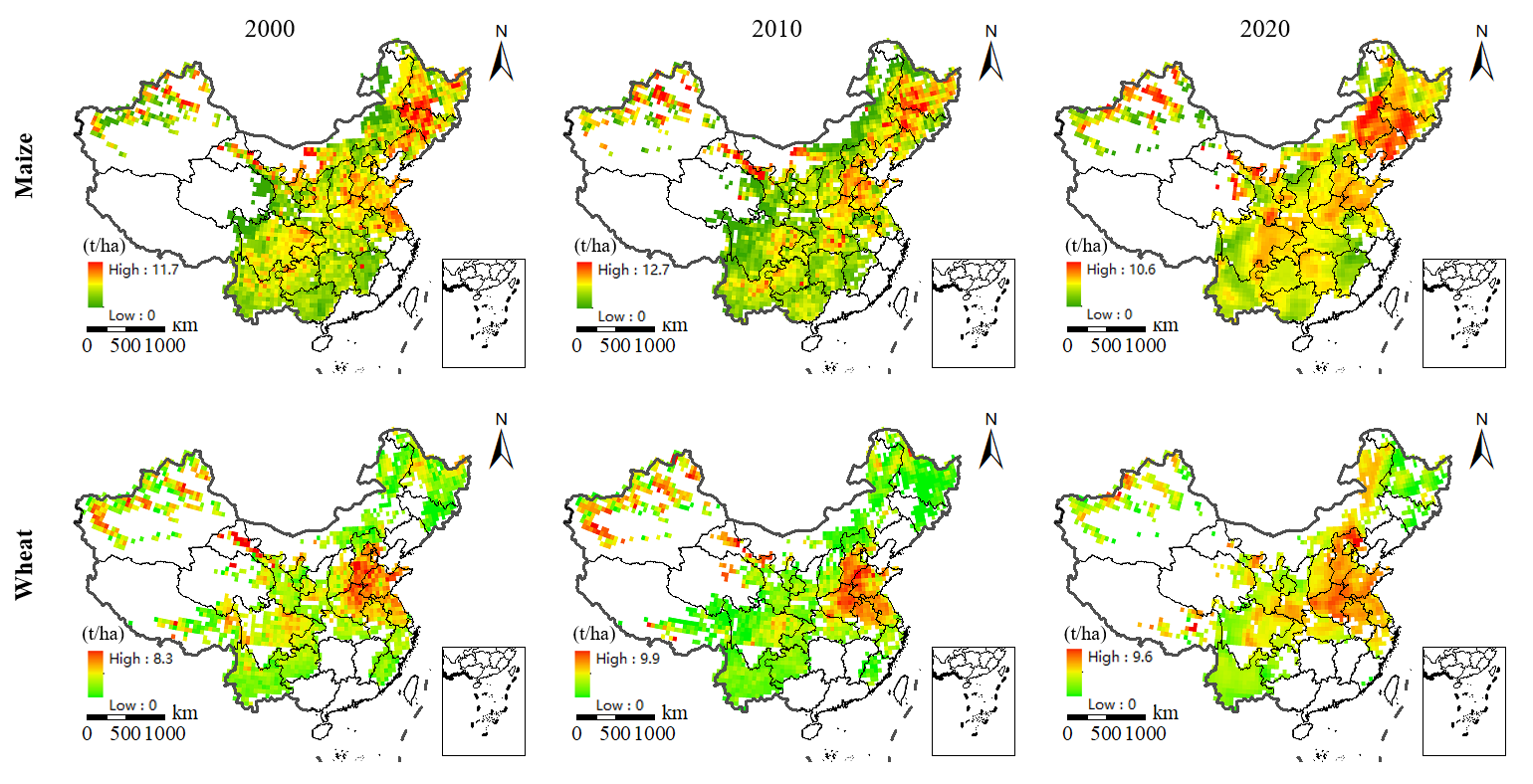


**Figure S3. Actual yield of maize and wheat in China in 2000, 2010 and 2020.**


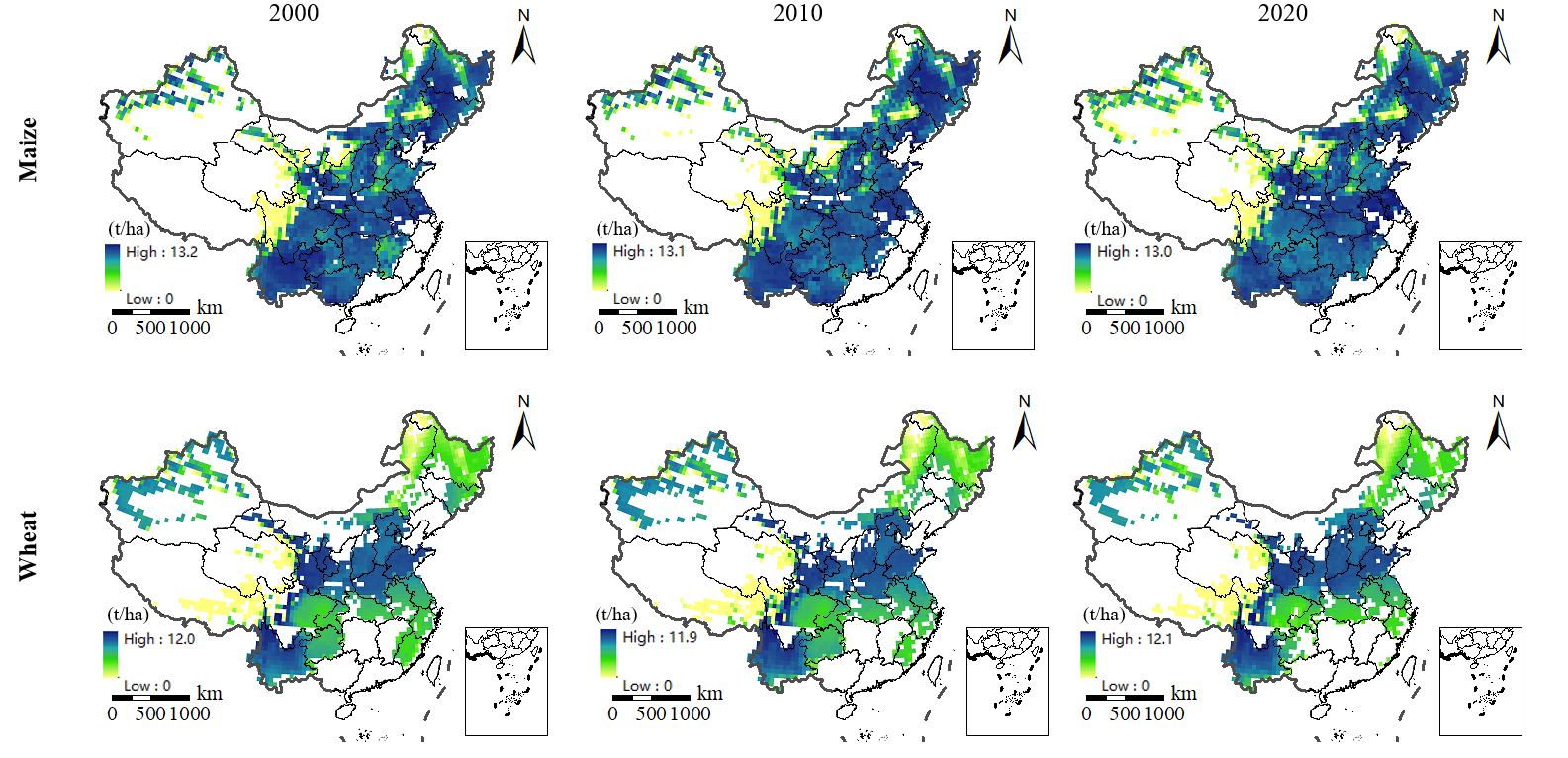


**Figure S4. Simulated climate resource-driven yield (CRY) of maize and wheat in 2000, 2010 and 2020.**


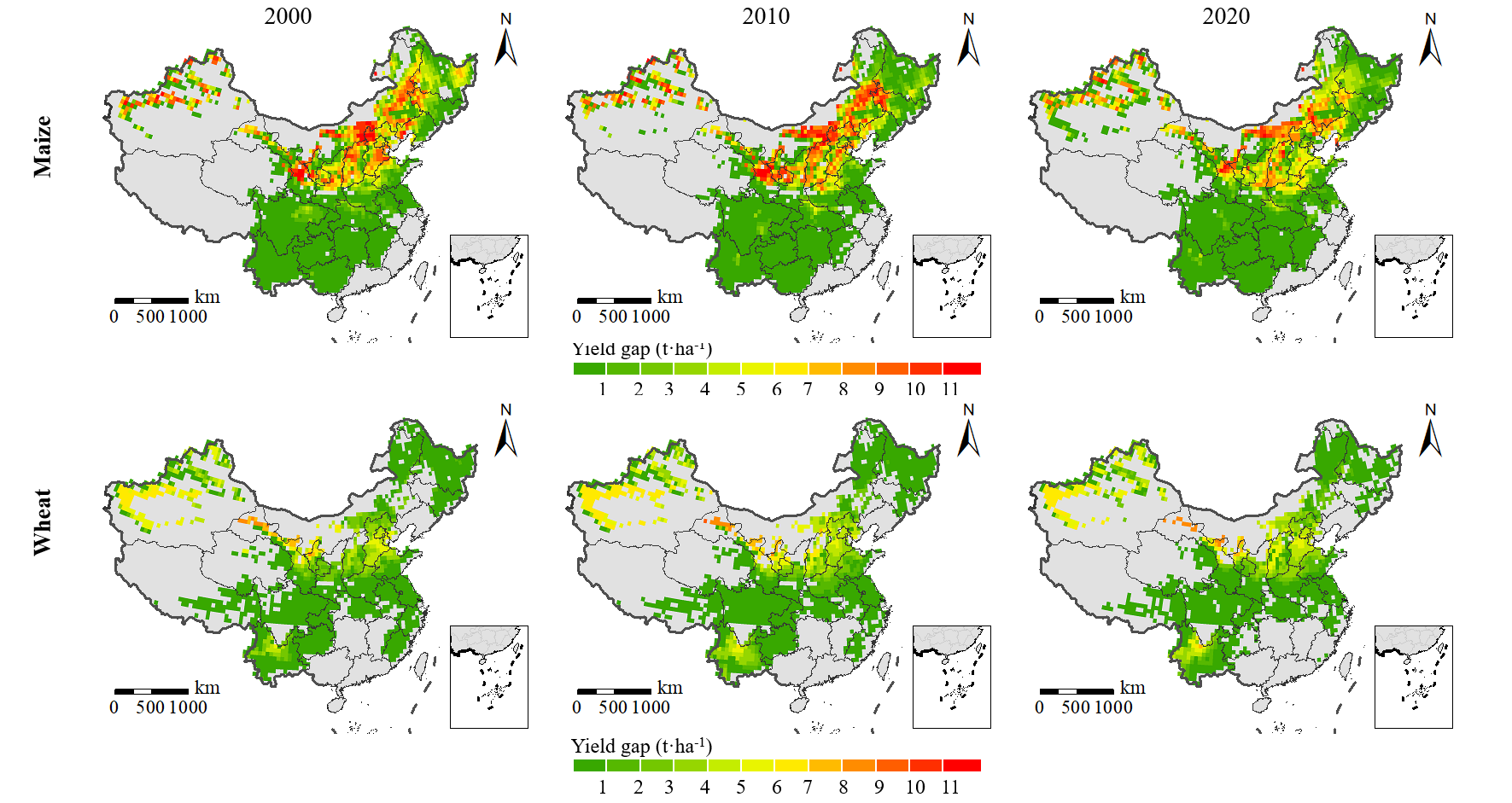


**Figure S5. Gap between climate resource-driven yield (CRY) and climate-water resource-driven yield (RY) in 2000, 2010, and 2020.**


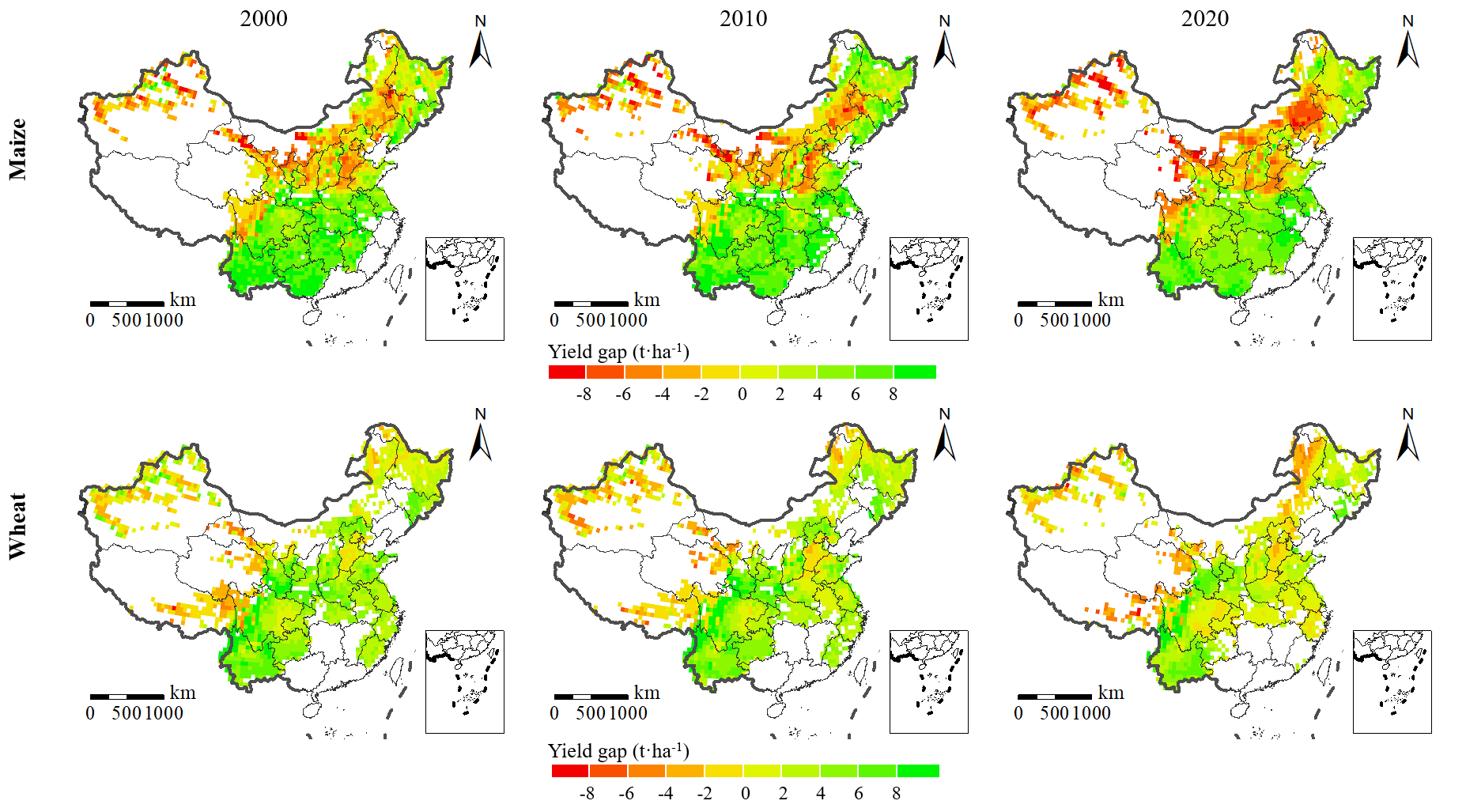
**Figure S6. Gap between climate-water resource-driven yield (RY) and actual yield in 2000, 2010, and 2020.**


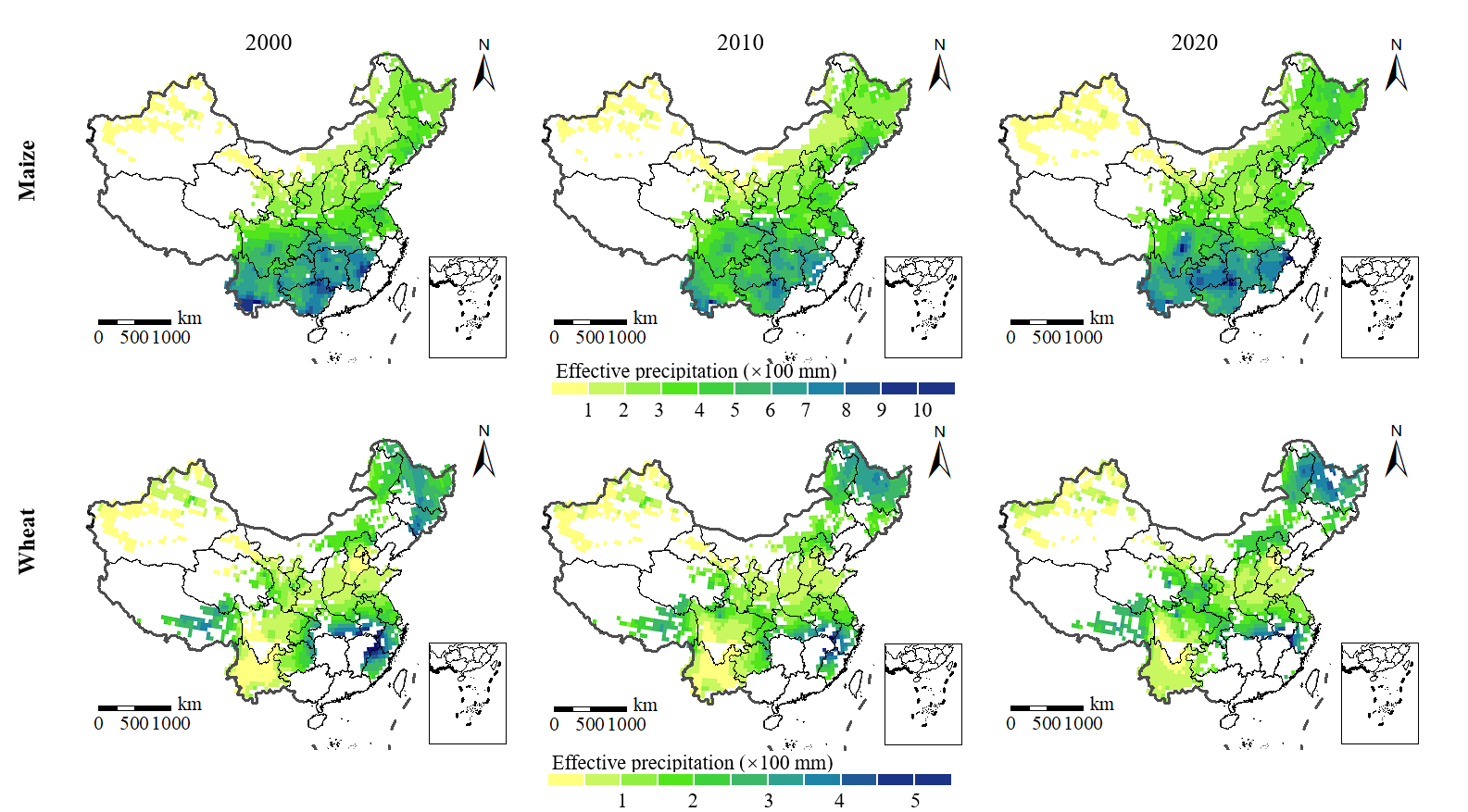


**Figure S7. Effective precipitation during whole growth period of maize and wheat in 2000, 2010 and 2020.**


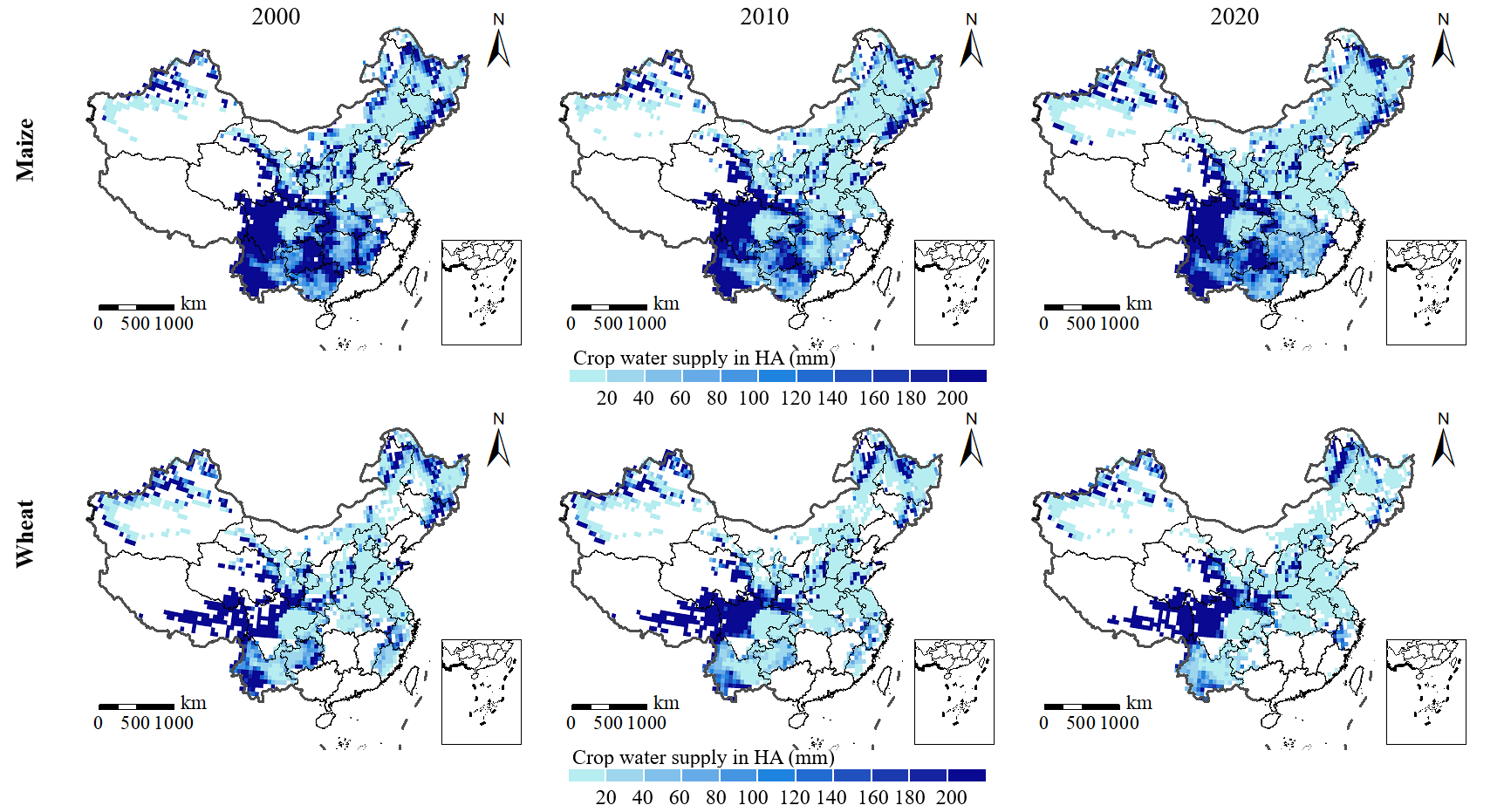


**Figure S8. Crop water supply (CWS) in maize and wheat harvest area in 2000, 2010 and 2020.**

Supplementary Tables

**Table S1. Details of the peer-reviewed published papers on the calibration and simulation of maize and wheat across China**

| Crop | Region | Province | Reference |
| --- | --- | --- | --- |
| Maize | NEC | HL, JL, LN | Gao et al. (2019); Gao et al. (2020); Li et al. (2022); Liu et al. (2013); Liu et al. (2012); Lv et al. (2015); Su et al. (2021); Yang et al. (2015); Zhang et al. (2018); Zhang et al. (2022); Zhao et al. (2023); Zhao et al. (2016); Zhao et al. (2020); Zhao et al. (2018); Zhao and Yang (2018, 2019); Zhu et al. (2022) |
|  | NC | BJ, TJ, HE, HA, NM, SX, SD | Gao et al. (2019); Gao et al. (2020); Liu et al. (2010); Wang et al. (2012); Yang et al. (2015); Zhang et al. (2018); Zhang et al. (2022); Zhao et al. (2020); Zhao et al. (2018); Zhao and Yang (2018, 2019) |
|  | NWC | XJ, GS, NX, SN, QH | Gao et al. (2019); Gao et al. (2020); Zhang et al. (2018); Zhang et al. (2022); Zhao and Yang (2019) |
|  | EC | SH, JS, ZJ, AH, FJ, JX | Zhang et al. (2018); Zhang et al. (2022) |
|  | CC | HA, HB, HN | Zhang et al. (2018); Zhang et al. (2022) |
|  | SWC | CQ, SC, GZ, YN, XZ | Zhang et al. (2018); Zhang et al. (2022); Zhao et al. (2020); Zhao et al. (2018); Zhao and Yang (2018, 2019) |
|  | SC | GX, GD | Zhang et al. (2018); Zhang et al. (2022) |
| Wheat | NEC | HL, JL, LN | Yang et al. (2015); Gao et al. (2020) |
|  | NC | BJ, TJ, HE, HA, NM, SX, SD | Gao et al. (2019); Li et al. (2014); Liu et al. (2010); Sun et al. (2018a, b); Sun et al. (2019); Yang et al. (2015); Zhang et al. (2022); Gao et al. (2020) |
|  | NWC | XJ, GS, NX, SN, QH | Sun et al. (2018b); Sun et al. (2019); Gao et al. (2020) |
|  | EC | SH, JS, ZJ, AH, FJ, JX | Sun et al. (2018a, b); Sun et al. (2019); Yang et al. (2015); Zhang et al. (2022) |
|  | CC | HA, HB, HN | Sun et al. (2018a, b); Sun et al. (2019); Yang et al. (2015); Zhang et al. (2022b) |
|  | SWC | CQ, SC, GZ, YN, XZ | Sun et al. (2018a, b); Sun et al. (2019); Yang et al. (2015); Zhang et al. (2022) |
|  | SC | GX, GD | Sun et al. (2018a, b) |

Note: NEC, NC, NWC, EC, CC, SWC, and SC represent Northeast China, North China, Northwest China, East China, Central China, Southwest China, and South China.

**Table S2. Simulation levels for resource-driven yield**

| Simulation levels | Resource dimensions | Determining factor |
| --- | --- | --- |
| Climate resource-driven yield (CRY) | Climate, soil | Radiation-temperature-precipitation-full irrigation-soil |
| Climate-water resource driven yield (RY) | Climate, water, soil | Radiation-temperature-precipitation-limited irrigation-soil |

**Table S3. Irrigation setting in APSIM in the simulation of RY**

| Water resource status | Description | Irrigation Setting |
| --- | --- | --- |
| CWA>1 | Local water resources are abundant enough to meet the crop water demand. | Full irrigation without irrigation water limitation, and automatic irrigation would be carried out once fraction of available soil water less than 0.75. |
| 0.5 < CWA ≤1 | Local water resources are abundant but not enough to meet the crop water demand. | Constrained irrigation with total available water equal to CWS, and automatic irrigation would be carried out once fraction of available soil water less than 0.75. |
| CWA ≤ 0.5 | Local water resources are limited and insufficient to meet the crop water demand. | Constrained irrigation with total available water equal to CWS, and automatic irrigation would be carried out once fraction of available soil water less than 0.5. |

Note: CWA and CWS represent crop water availability and crop water supply, respectively.

References

Allen, R., Pereira, L., Raes, D., Smith, M., Allen, R. G., Pereira, L. S. (1998). Crop evapotranspiration: guidelines for computing crop water requirements, FAO irrigation and drainage paper 56. FAO, 56.

Gao, J., Yang, X., Zheng, B., Liu, Z., Zhao, J., Sun, S., Li, K., and Dong, C. (2019). Effects of climate change on the extension of the potential double cropping region and crop water requirements in Northern China. Agricultural and Forest Meteorology 268, 146-155.

Gao, J., Yang, X., Zheng, B., Liu, Z., Zhao, J., and Sun, S. (2020). Does precipitation keep pace with temperature in the marginal double-cropping area of northern China? European Journal of Agronomy 120, 126126.

Li, E., Zhao, J., Pullens, J. W. M., and Yang, X. (2022). The compound effects of drought and high temperature stresses will be the main constraints on maize yield in Northeast China. Science of The Total Environment 812, 152461.

Li, K., Yang, X., Liu, Z., Zhang, T., Lu, S., and Liu, Y. (2014). Low yield gap of winter wheat in the North China Plain. European Journal of Agronomy 59, 1-12.

Liu, Y., Wang, E., Yang, X., and Wang, J. (2010). Contributions of climatic and crop varietal changes to crop production in the North China Plain, since 1980s. Global Change Biology 16, 2287-2299.

Liu, Z., Hubbard, K. G., Lin, X., and Yang, X. (2013). Negative effects of climate warming on maize yield are reversed by the changing of sowing date and cultivar selection in Northeast China. Global Change Biology 19, 3481-3492.

Liu, Z., Yang, X., Hubbard, K. G., and Lin, X. (2012). Maize potential yields and yield gaps in the changing climate of northeast China. Global Change Biology 18, 3441-3454.

Lv, S., Yang, X., Lin, X., Liu, Z., Zhao, J., Li, K., Mu, C., Chen, X., Chen, F., and Mi, G. (2015). Yield gap simulations using ten maize cultivars commonly planted in Northeast China during the past five decades. Agricultural and Forest Meteorology 205, 1-10.

Peng, S. (2022). 1-km monthly potential evapotranspiration dataset for China (1901-2024). National Tibetan Plateau / Third Pole Environment Data Center.

Su, Z., Liu, Z., Bai, F., Zhang, Z., Sun, S., Huang, Q., Liu, T., Liu, X., and Yang, X. (2021). Cultivar selection can increase yield potential and resource use efficiency of spring maize to adapt to climate change in Northeast China. Journal of Integrative Agriculture 20, 371-382.

Sun, S., Yang, X., Lin, X., Sassenrath, G. F., and Li, K. (2018a). Climate-smart management can further improve winter wheat yield in China. Agricultural Systems 162, 10-18.

Sun, S., Yang, X., Lin, X., Sassenrath, G. F., and Li, K. (2018b). Winter Wheat Yield Gaps and Patterns in China. Agronomy Journal 110, 319-330.

Sun, S., Yang, X., Lin, X., Zhao, J., Liu, Z., Zhang, T., and Xie, W. (2019). Seasonal variability in potential and actual yields of winter wheat in China. Field Crops Research 240, 1-11.

Wang, J., Wang, E., Yang, X., Zhang, F., and Yin, H. (2012). Increased yield potential of wheat-maize cropping system in the North China Plain by climate change adaptation. Climatic Change 113, 825-840.

Yang, X., Chen, F., Lin, X., Liu, Z., Zhang, H., Zhao, J., Li, K., Ye, Q., Li, Y., Lv, S., Yang, P., Wu, W., Li, Z., Lal, R., and Tang, H. (2015). Potential benefits of climate change for crop productivity in China. Agricultural and Forest Meteorology 208, 76-84.

Yin, X. G., Jabloun, M., Olesen, J. E., Öztürk, I., Wang, M., and Chen, F. (2016). Effects of climatic factors, drought risk and irrigation requirement on maize yield in the Northeast Farming Region of China. The Journal of Agricultural Science 154, 1171-1189.

Zhang, T., Yue, X., Li, T., Unger, N., and Yang, X. (2018). Climate effects of stringent air pollution controls mitigate future maize losses in China. Environmental Research Letters 13, 124011.

Zhang, Z., Sun, S., Zhang, F., Guo, S., Guo, E., Liu, Z., Zhao, J., Zhao, C., Li, T., and Yang, X. (2022). Using estimated radiation in crop models amplified the negative impacts of climate variability on maize and winter wheat yields in China. Agricultural and Forest Meteorology 318, 108914.

Zhao, J., Liu, Z., Lv, S., Lin, X., Li, T., and Yang, X. (2023). Changing maize hybrids helps adapt to climate change in Northeast China: revealed by field experiment and crop modelling. Agricultural and Forest Meteorology 342.

Zhao, J., Yang, X., Liu, Z., Lv, S., Wang, J., and Dai, S. (2016). Variations in the potential climatic suitability distribution patterns and grain yields for spring maize in Northeast China under climate change. Climatic Change 137, 29-42.

Zhao, J., Yang, X., Liu, Z., Pullens, J. W. M., Chen, J., Marek, G. W., Chen, Y., Lv, S., and Sun, S. (2020). Greater maize yield improvements in low/unstable yield zones through recommended nutrient and water inputs in the main cropping regions, China. Agricultural Water Management 232, 106018.

Zhao, J., Yang, X., and Sun, S. (2018). Constraints on maize yield and yield stability in the main cropping regions in China. European Journal of Agronomy 99, 106-115.

Zhao, J., and Yang, X. (2018). Distribution of high-yield and high-yield-stability zones for maize yield potential in the main growing regions in China. Agricultural and Forest Meteorology 248, 511-517.

Zhao, J., and Yang, X. (2019). Spatial patterns of yield-based cropping suitability and its driving factors in the three main maize-growing regions in China. International Journal of Biometeorology 63, 1659-1668.

Zhu, G., Liu, Z., Qiao, S., Zhang, Z., Huang, Q., Su, Z., and Yang, X. (2022). How could observed sowing dates contribute to maize potential yield under climate change in Northeast China based on APSIM model. European Journal of Agronomy 136, 126511.
